# Bob1 maintains T follicular helper cells for long-term humoral immunity

**DOI:** 10.1038/s42003-024-05827-0

**Published:** 2024-02-15

**Authors:** Masahiro Yanagi, Ippei Ikegami, Ryuta Kamekura, Tatsuya Sato, Taiki Sato, Shiori Kamiya, Kosuke Murayama, Sumito Jitsukawa, Fumie Ito, Akira Yorozu, Miho Kihara, Takaya Abe, Hiromi Takaki, Koji Kawata, Katsunori Shigehara, Satsuki Miyajima, Hirotaka Nishikiori, Akinori Sato, Noritsugu Tohse, Ken-ichi Takano, Hirofumi Chiba, Shingo Ichimiya

**Affiliations:** 1 Department of Human Immunology, Research Institute for Frontier Medicine, Sapporo Medical University School of Medicine, Sapporo, 060-8556 Japan; 2 Department of Respiratory Medicine and Allergology, Sapporo Medical University School of Medicine, Sapporo, 060-8556 Japan; 3 Department of Otolaryngology-Head and Neck Surgery, Sapporo Medical University School of Medicine, Sapporo, 060-8556 Japan; 4 Department of Cellular Physiology and Signal Transduction, Sapporo Medical University School of Medicine, Sapporo, 060-8556 Japan; 5 Laboratory for Animal Resources and Genetic Engineering, RIKEN Center for Biosystems Dynamics Research, Kobe, 650-0047 Japan; 6 Department of Rehabilitation, Faculty of Healthcare and Science, Hokkaido Bunkyo University, Eniwa, 061-1449 Japan

**Keywords:** Antibodies, Immunological memory, Germinal centres, Antibodies, Follicular T-helper cells

## Abstract

Humoral immunity is vital for host protection, yet aberrant antibody responses can trigger harmful inflammation and immune-related disorders. T follicular helper (Tfh) cells, central to humoral immunity, have garnered significant attention for unraveling immune mechanisms. This study shows the role of B-cell Oct-binding protein 1 (Bob1), a transcriptional coactivator, in Tfh cell regulation. Our investigation, utilizing conditional Bob1-deficient mice, suggests that Bob1 plays a critical role in modulating inducible T-cell costimulator expression and cellular respiration in Tfh cells. This regulation maintains the long-term functionality of Tfh cells, enabling their reactivation from central memory T cells to produce antibodies during recall responses. In a bronchial asthma model induced by house dust mite (HDM) inhalation, Bob1 is observed to enhance HDM-specific antibodies, including IgE, highlighting its pivotal function in Tfh cell regulation. Further exploration of Bob1-dependent mechanisms in Tfh cells holds promise for governing protective immunity and addressing immune-related disorders.

## Abstract (web summary)

Bob1 manages ICOS and cellular respiration in Tfh cells, ensuring the sustained generation of specific humoral responses. This likely contributes to persistent inflammation resulting from recurring allergen exposure.

## Introduction

Adaptive humoral immunity is a cardinal arm of the defense system that protects the host from pathogens. However, dysfunction of the antigen-specific antibody responses can lead to the development of a variety of immune-related disorders, including allergies and autoimmunity^1,2^. The production of high-affinity antibodies by plasma cells and memory B cells generally requires the cognate interactions between B cells and T follicular helper (Tfh) cells within lymphoid tissues^3^. On this basis, the pathological relationship between B cells and Tfh cells has been a key focus of recent research on various immune-related disorders^4,5^. Given the critical importance of Tfh cells in modulating specific humoral responses, the regulatory mechanism of Tfh cells in the initial and recall phases is a highly clinically relevant topic^6,7^.

Tfh cells are characterized by the production of B-cell cytokines such as IL-4 and IL-21 and the expression of cell-surface regulatory molecules such as C-X-C chemokine receptor type 5 (CXCR5), programmed cell death-1 (PD-1), and inducible T-cell costimulator (ICOS; ref. ^6^). Tfh cells are primarily differentiated from naive CD4^+^ T cells and are equipped with exclusive machinery for bimodal cell-fate decisions, which are coordinated by reciprocally antagonistic transcription factors such as B lymphocyte-induced maturation protein 1 (Blimp-1) and B-cell lymphoma 6 (Bcl6), the master regulator of Tfh cells^8,9^. Accumulating evidence has shown that human and mouse Tfh cells preferentially express a transcriptional coactivator of B-cell Oct-binding protein-1 (Bob1), in which Oct represents a DNA-binding molecule specific to the octamer motif with an 8-bp element, ATTTGCAT^10–12^. Bob1, also known as POU class 2 homeobox associating factor 1 (Pou2af1), Oct coactivator from B cells (Oca-B), or Oct-binding factor 1 (Obf-1), can be induced in activated CD4^+^ T cells through stimulation of the T cell receptor, suggesting a role for Bob1 in effector CD4^+^ T cell subsets^13–15^. Bob1 was originally identified as a proline-rich transcriptional coactivator that binds to a ubiquitously expressed (Pou2f1, Oct-1) or lymphocyte-specific (Pou2f2, Oct-2) octamer-binding transcription factor, through which immunoglobulin gene expression is strictly controlled in B cells^16–19^. The systemic deletion of the *Pou2af1* gene encoding Bob1 has been shown to impair the formation of germinal centers (GCs) in lymphoid tissues, leading to functional defects in the generation of antigen-specific antibodies and memory B cells, demonstrating the pivotal role of Bob1 in the establishment of specific humoral immunity^20,21^. Similar to other functional molecules, including Bcl6, CXCR5, and basic helix-loop-helix family member e40 (Bhlhe40), the Bob1 coactivator is shared by B cells and Tfh cells^8,22^. Consequently, the biological significance of Bob1 in T cells has been studied by assessing various immune responses in T-cell-specific Bob1-deficient mice, in which B cells retain Bob1 expression^23,24^. However, the mechanism through which Bob1 contributes to the functional control of Tfh cells in protective immunity remains to be addressed^25^.

In this study, we established CD4-Cre Bob1 knockout (CD4^Cre/+^Bob1^fl/fl^) mice, by ablating the entire gene encoding Bob1, as well as FoxP3^GFP/DTR^CD4^Cre/+^Bob1^fl/fl^ mice, to investigate Tfh cell regulation in relation to T follicular regulatory cells (Tfr cells), because the GC response is dependent on the balance between Tfh cells and Tfr cells^5,26^. Studies using T-dependent antigens demonstrated that Bob1 controls the maintenance and reactivation potential of Tfh cells from the central memory T-cell (Tcm) pool, thereby sustaining specific humoral responses. This is likely due to the regulatory mechanism of Bob1 in cellular respiration as well as the expressions of ICOS and IL-4 in Tfh cells^6,27^. Moreover, experiments using an allergic asthma model induced by the repetitive inhalation of house dust mite (HDM), the most common in-house allergen in asthma, revealed the role of Bob1 in severe airway inflammation and the promotion of HDM-specific antibody responses including IgE. Collectively, Bob1 possesses the fundamental capacity to regulate the long-term responses of Tfh cells and ensure lasting antigen-specific antibody responses.

## Results

### Bob1 regulates Tfh cells to establish adaptive humoral response

**Fig. 1:**
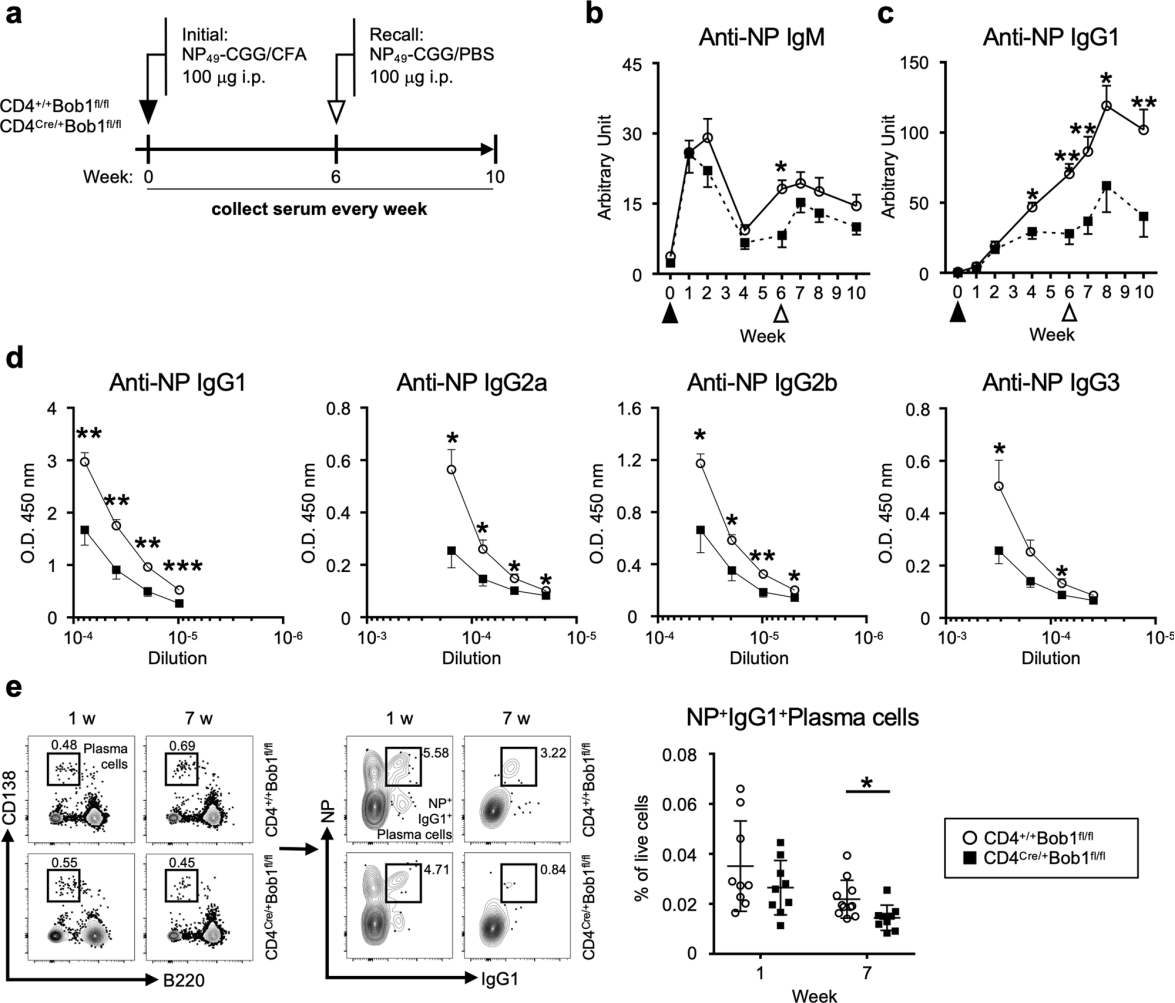
Bob1 regulates Tfh cells to produce antigen-specific class-switched antibodies. **a** Experimental protocol for the immunization of CD4^Cre/+^Bob1^fl/fl^ mice and control CD4^+/+^Bob1^fl/fl^ mice. NP_49_-CGG was administered intraperitoneally with CFA on day 0 and with PBS on day 42. Sera were collected from the tail vein every week until day 70 and examined by ELISA using NP_27_-BSA. **b**, **c** Time courses of NP-specific antibodies in sera. **b** NP-specific IgM, *P = 0.0148. **c** NP-specific IgG1, *P = 0.0207, **P = 0.0030, **P = 0.0070, *P = 0.0274, **P = 0.0070, in order from left to right. **d** Levels of NP-specific IgG subclasses in sera on day 49 as examined in different dilutions. NP-specific IgG1, **P = 0.0011, **P = 0.0011, **P = 0.0011, ***P = 0.0006; NP-specific IgG2a, *P = 0.0148, *P = 0.0148, *P = 0.0281, *P = 0.0221; NP-specific IgG2b, *P = 0.0499, *P = 0.0463, **P = 0.0096, *P = 0.0379; NP-specific IgG3, *P = 0.0499, *P = 0.0379, in order from left to right dilution points. **e** Representative flow cytometric profiles and graphs of NP^+^IgG1^+^ plasma cells (NP^+^IgG1^+^B220^lo^CD138^hi^) in spleens. *P = 0.0132. Data represent the mean ± SEM in (b-d) or the mean ± SD in (e) in 8-11 mice per group. Statistical significance was analyzed by the Mann-Whitney *U*-test; *P < 0.05, **P < 0.01, ***P < 0.001. Data shown in (b-e) are from CD4^+/+^Bob1^fl/fl^ mice (open circle) and CD4^Cre/+^Bob1^fl/fl^ mice (closed square). Similar results were obtained across three independent experiments.

To examine the role of Bob1 in the regulation of Tfh cells, we generated mice with a floxed allele covering the entire *Pou2af1* gene, which encodes Bob1, using CRISPR-Cas9 genome editing technology (Bob1^fl/+^ mice; Supplementary Fig. 1a–c). Subsequently, Bob1^fl/+^ mice were crossed with CD4^Cre/+^ mice, which express Cre recombinase under the transcriptional control of the *Cd4* promoter element, to obtain CD4^Cre/+^Bob1^fl/fl^ mice. In a pre-immune state, the levels of immature and mature immune cells residing in the thymus, bone marrow, and spleen of CD4^Cre/+^Bob1^fl/fl^ mice were comparable to those of control CD4^+/+^Bob1^fl/fl^ mice (Supplementary Fig. 2a–g). Using these mice, we initially analyzed the immune responses against 4-hydroxy-3-nitrophenylacetyl (NP), a T-cell-dependent antigen. Mice were intraperitoneally administered NP-conjugated chicken gamma globulin (NP-CGG) admixed with complete Freund’s adjuvant (CFA), and, 6 weeks later, they were intraperitoneally rechallenged with NP-CGG in phosphate-buffered saline (PBS; Fig. 1a). Serum samples were collected weekly from tail veins throughout the observation period, which lasted until day 70 in this experiment, to measure the levels of NP-specific antibodies. The levels of NP-specific IgM in CD4^Cre/+^Bob1^fl/fl^ mice and control CD4^+/+^Bob1^fl/fl^ mice showed similar changes (Fig. 1b). However, the levels of NP-specific IgG1 in CD4^Cre/+^Bob1^fl/fl^ mice remained low compared to those in control CD4^+/+^Bob1^fl/fl^ mice (Fig. 1c). Similarly, the serum titers of other NP-specific IgG subclasses, including IgG2a, IgG2b, and IgG3, were generally lower in CD4^Cre/+^Bob1^fl/fl^ mice compared to those in CD4^+/+^Bob1^fl/fl^ mice (Fig. 1d), suggesting a possible role of Bob1 in the regulation of adaptive humoral immunity. The reduced level of IgG1^+^ plasma cells specifically binding to NP further indicated aberrant humoral responses in CD4^Cre/+^Bob1^fl/fl^ mice (Fig. 1e). Consistent results were observed in experiments using sheep red blood cells (SRBCs), suggesting that CD4^Cre/+^Bob1^fl/fl^ mice were inefficient inducers of specific class-switched antibody responses against foreign antigens (Supplementary Fig. 3a–c). Studies of FoxP3^GFP/DTR^ mice to separately identify Tfh cells and Tfr cells showed that Tfh cells demonstrated high expression of *Pou2af1* (Bob1), while Tfr cells, regulatory T cells (Treg cells), and conventional T cells (Tconv cells) expressed *Pou2af1* at very low levels, and *Pou2f2* (Oct-2) was dominant in Tfh cells (Supplementary Fig. 4a–d; refs. ^26,28^). In vitro studies of naive CD4^+^ T cells from FoxP3^GFP/DTR^CD4^Cre/+^Bob1^fl/fl^ mice and control FoxP3^GFP/DTR^CD4^+/+^Bob1^fl/fl^ mice suggested that their differentiation processes into effector CD4^+^ T cells, such as Th1, Th2, and Th17 cells, were unlikely to be affected by the absence of Bob1 (Supplementary Fig. 4e). We also found that Bob1 was abundant in Tfh cells (CD45RA^-^CXCR5^+^PD-1^+^) and, to a lesser extent, in inter-follicular-type Tfh cells (CD45RA^-^CXCR5^lo^PD-1^lo^) in human tonsillar lymphocytes (Supplementary Fig. 4f). Collectively, these findings suggest that Tfh cells are possibly dependent on Bob1 for humoral adaptation with isotype-switched antibodies.

### Bob1 regulates Tfh cells irrespective of Tfr cells

**Fig. 2:**
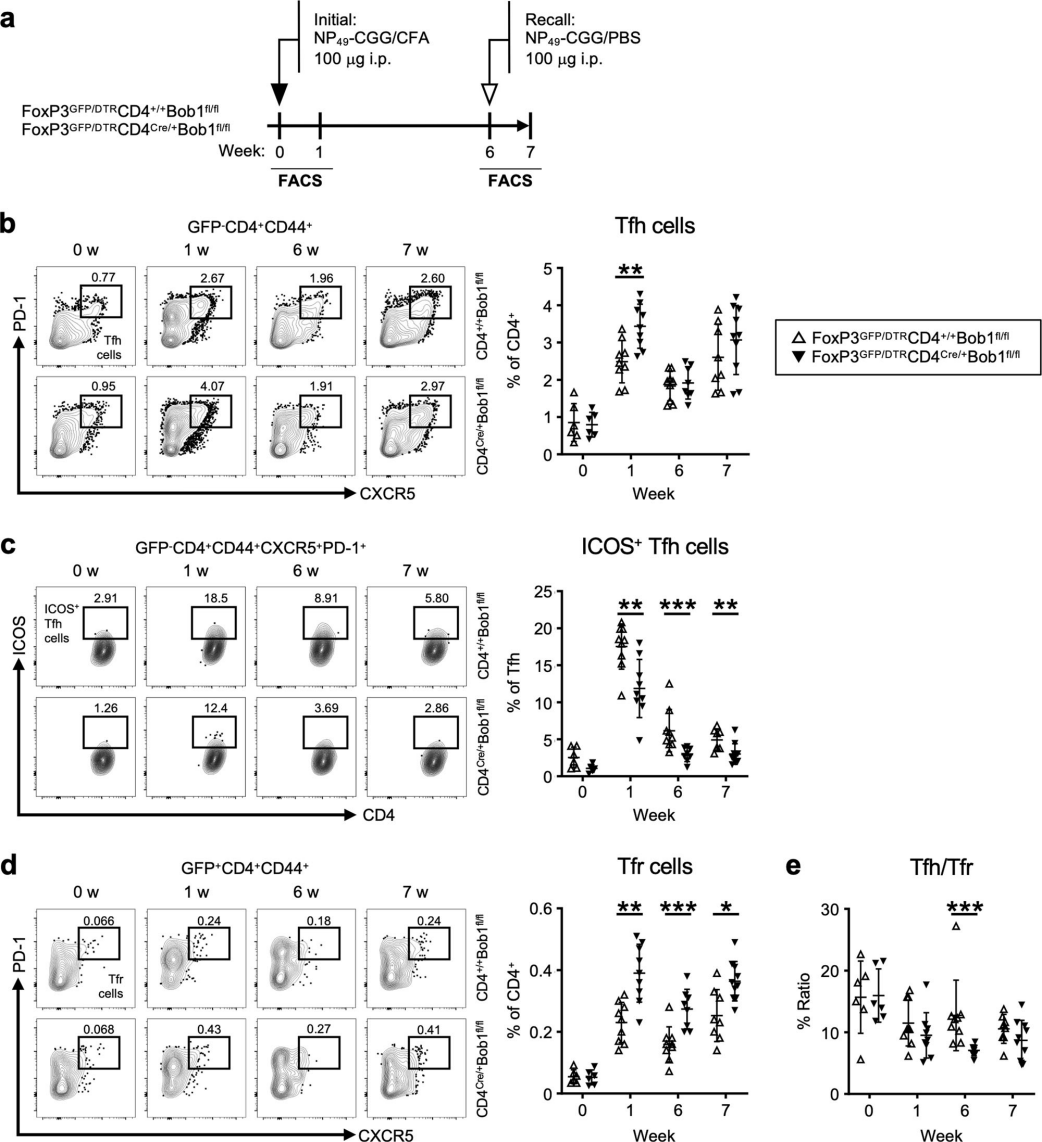
Bob1 regulates the function of ICOS^+^ Tfh cells. **a** Experimental protocol for immunization of FoxP3^GFP/DTR^CD4^Cre/+^Bob1^fl/fl^ mice and FoxP3^GFP/DTR^CD4^+/+^Bob1^fl/fl^ mice as controls. NP_49_-CGG was administered intraperitoneally with CFA on day 0 and with PBS on day 42. Spleen cells were analyzed by flow cytometry on days 0, 7, 42, and 49. **b**–**d** Representative flow cytometric profiles and graphs of CD4^+^ T cell subsets. **b** Tfh cells (GFP^-^B220^-^CD4^+^CD44^+^CXCR5^+^PD-1^+^), **P = 0.0040. **c** ICOS^+^ Tfh cells, **P = 0.0055, ***P = 0.0006, **P = 0.0092, in order from left to right in graphs of the % of ICOS^+^ Tfh cells among the total Tfh cells. **d** Tfr cells (GFP^+^B220^-^CD4^+^CD44^+^CXCR5^+^PD-1^+^), **P = 0.0014, ***P = 0.0009, *P = 0.0114, in order from left to right in graphs of the % of Tfr cells among the total CD4^+^ T cells. **e** Ratio of Tfh cells per Tfr cells based on the results from (**b**) and (**d**). ***P < 0.0003. Data represent the mean ± SD of 6–10 mice per group. Statistical significance was analyzed by the Mann-Whitney *U*-test; *P < 0.05, **P < 0.01, ***P < 0.001. Data in (**b**–**d**) are shown from FoxP3^GFP/DTR^CD4^+/+^Bob1^fl/fl^ mice (open triangle) and FoxP3^GFP/DTR^CD4^Cre/+^Bob1^fl/fl^ mice (closed triangle). Similar results were obtained across three independent experiments.

To further investigate the regulation of adaptive humoral immunity by Bob1-associated Tfh-cell function, we analyzed immune responses in FoxP3^GFP/DTR^CD4^Cre/+^Bob1^fl/fl^ mice and control FoxP3^GFP/DTR^CD4^+/+^Bob1^fl/fl^ mice, focusing on the functional features of Tfh cells and Tfr cells. Using the same immunization protocol as that outlined in Fig. 1, we observed a transient elevation in the proportion of Tfh cells after the initial administration of NP-CGG (Fig. 2a, b, Supplementary Fig. 5a). However, ICOS-expressing Tfh cells were consistently reduced in FoxP3^GFP/DTR^CD4^Cre/+^Bob1^fl/fl^ mice (Fig. 2c). The low expression level of ICOS in CD4^Cre/+^Bob1^fl/fl^ Tfh cells may be one of the mechanisms underlying their malfunction, as ICOS expression in Tfh cells is necessary for the class switch recombination of immunoglobulin genes against previously encountered antigens^29,30^. Further analysis revealed the increased level of Tfr cells in FoxP3^GFP/DTR^CD4^Cre/+^Bob1^fl/fl^ mice, which resulted in a decreased Tfh/Tfr ratio (Fig. 2d, e). Following these results, we next examined the effect of Tfr cells on Tfh cell function in FoxP3^GFP/DTR^CD4^Cre/+^Bob1^fl/fl^ mice to better understand the roles of Bob1 in the regulation of Tfh cells. To evaluate the role of Tfr cells in the regulation of Tfh cell function, we performed in vivo removal of Tfr cells by intraperitoneally injecting diphtheria toxin after the administration of NP-CGG (Supplementary Fig. 6a, b). Consistent with the results shown in Fig. 2, the NP-specific IgM responses in FoxP3^GFP/DTR^CD4^Cre/+^Bob1^fl/fl^ mice were similar to those in FoxP3^GFP/DTR^CD4^+/+^Bob1^fl/fl^ mice even after the depletion of Tfr cells (Supplementary Fig. 6c). However, the level of NP-specific IgG1 remained low in FoxP3^GFP/DTR^CD4^Cre/+^Bob1^fl/fl^ mice compared to FoxP3^GFP/DTR^CD4^+/+^Bob1^fl/fl^ mice (Supplementary Fig. 6c). Notably, the removal of Tfr cells unlikely influenced the expression level of ICOS on Tfh cells in FoxP3^GFP/DTR^CD4^Cre/+^Bob1^fl/fl^ mice, which was still lower than that in FoxP3^GFP/DTR^CD4^+/+^Bob1^fl/fl^ mice (Supplementary Fig. 6d, e). Taken together, these findings indicate that Bob1 regulates the function of Tfh cells independently of Tfr cells.

### Bob1 characterizes unique gene signatures supporting functional Tfh cells

**Fig. 3:**
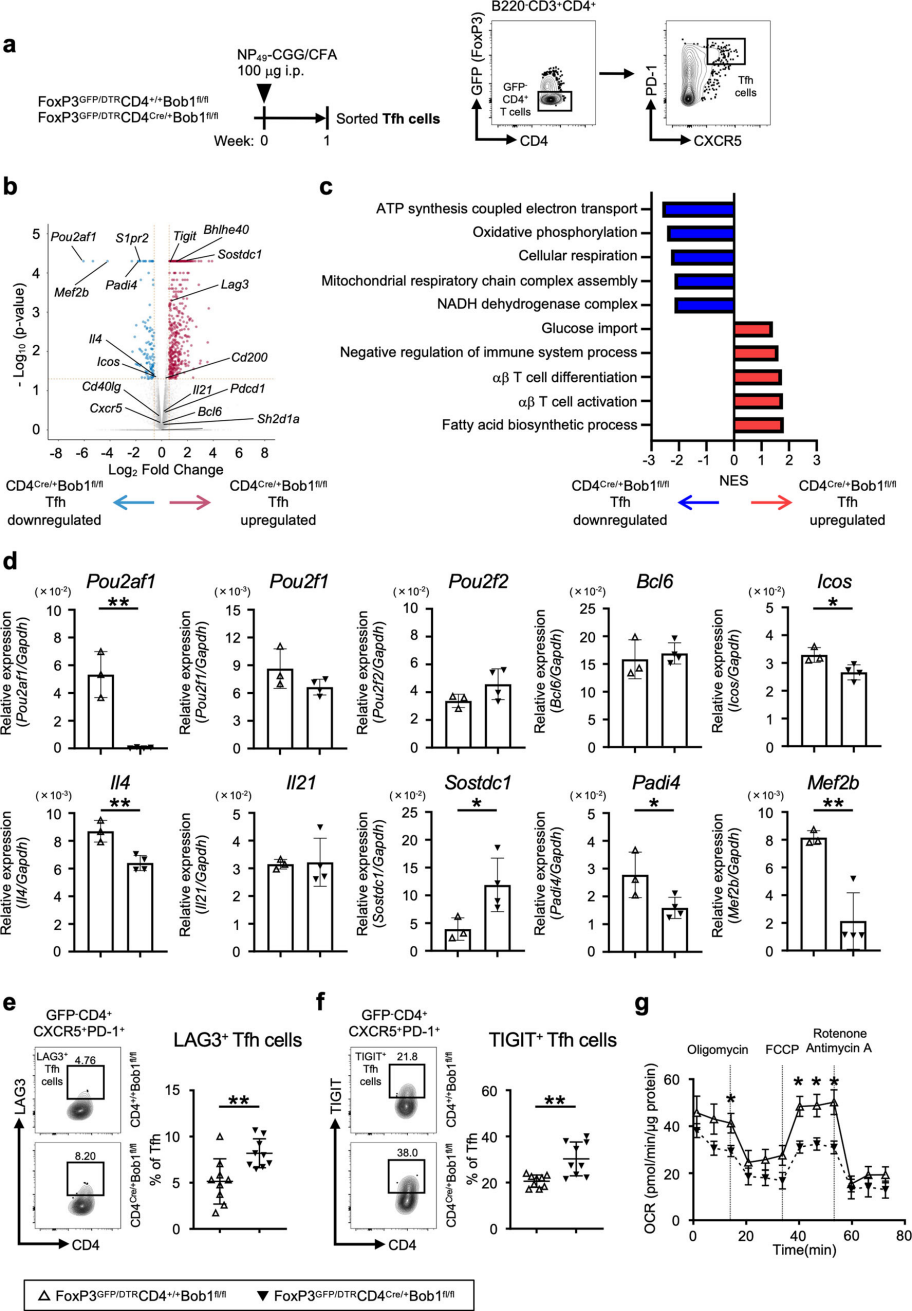
Gene signatures regulated by Bob1 in Tfh cells. **a** Experimental protocol for RNAseq analysis using FoxP3^GFP/DTR^CD4^Cre/+^Bob1^fl/fl^ mice and control FoxP3^GFP/DTR^CD4^+/+^Bob1^fl/fl^ mice to obtain CD4^Cre/+^Bob1^fl/fl^ Tfh cells and CD4^+/+^Bob1^fl/fl^ Tfh cells, respectively. NP_49_-CGG was administered intraperitoneally with CFA on day 0. As shown in the gating strategy, CD4^Cre/+^Bob1^fl/fl^ and CD4^+/+^Bob1^fl/fl^ Tfh cells (GFP^-^B220^-^CD3^+^CD4^+^CXCR5^+^PD-1^+^) were sorted from spleens on day 7 and subjected to RNAseq analysis. **b** Volcano plot showing differentially expressed genes (*P* < 0.05) with more than two-fold expression (log_2_ fold change > 0.5) in CD4^Cre/+^Bob1^fl/fl^ Tfh cells vs. CD4^+/+^Bob1^fl/fl^ Tfh cells. The red and blue dots indicate the upregulated and downregulated genes, respectively, in CD4^Cre/+^Bob1^fl/fl^ Tfh cells. **c** Gene set enrichment analysis (GSEA) of the transcriptomes of CD4^Cre/+^Bob1^fl/fl^ and CD4^+/+^Bob1^fl/fl^ Tfh cells. Gene sets include ATP synthesis coupled electron transport (GO: 0042773), oxidative phosphorylation (GO: 0006119), cellular respiration (GO: 0045333), mitochondrial respiratory chain complex assembly (GO: 0033108), NADH dehydrogenase complex (GO: 0030964), glucose import (GO: 0046323), negative regulation of immune system process; (GO: 0002683), αβ-T cell differentiation (GO: 0046632), αβ-T cell activation (GO: 0046631), and fatty acid biosynthetic process (GO: 0045723). Gene sets showed *P* < 0.05 and FDR < 0.25. NES, normalized enrichment score. **d** Relative mRNA expression levels of *Pou2af1*, *Pou2f1*, *Pou2f2*, *Bcl6*, *Icos*, *Il4, Il21, Sostdc1, Padi4*, and *Mef2b* in CD4^Cre/+^Bob1^fl/fl^ and CD4^+/+^Bob1^fl/fl^ Tfh cells examined by RT-qPCR analysis. *Pou2af1*, **P = 0.0012; *Icos*, *P = 0.0284; *Il4*, **P = 0.0058; *Sostdc1*, *P = 0.0462; *Padi4*, *P = 0.0478; *Mef2b*, **P = 0.0045. **e**, **f** Representative flow cytometric profiles and graphs of the expression of negative regulators in CD4^Cre/+^Bob1^fl/fl^ and CD4^+/+^Bob1^fl/fl^ Tfh cells as measured in (**a**). **e** Profiles of Lag3^+^ Tfh cells and graphs of the % of Lag3^+^ Tfh cells among the total Tfh cells. **P = 0.0056. **f** Profiles of Tigit^+^ Tfh cells and graphs of the % of Tigit^+^ Tfh cells among the total Tfh cells. **P = 0.0039. **g** Oxygen consumption rate (OCR) of CD4^Cre/+^Bob1^fl/fl^ and CD4^+/+^Bob1^fl/fl^ Tfh cells. The OCR was measured after supplementation with oligomycin (a complex V inhibitor), FCCP (a protonophore), and rotenone/antimycin A (complex I/III inhibitors). *P = 0.0303, *P = 0.0101, *P = 0.0101, *P = 0.0177, in order from left to right. RNAseq data in (**b**) were obtained from specimens of three experiments in each mouse group (*n* = 10–12 per experiment). Data from RT-qPCR analysis in (**d**) represents the mean ± SD by unpaired *t-*test (1 dot; *n* = 4–5), *P < 0.05, **P < 0.01. Graphs in (**e**, **f**) show the mean ± SD determined by the Mann-Whitney *U-*test (**e**, **f**, *n* = 9), **P < 0.01. Graphs in (**g**) show the mean ± SEM determined by the Mann–Whitney *U-*test (CD4^+/+^Bob1^fl/fl^ Tfh cells; *n* = 5, CD4^Cre/+^Bob1^fl/fl^ Tfh cells; *n* = 7). *P < 0.05. Data in (**d**–**f**) are shown from FoxP3^GFP/DTR^CD4^+/+^Bob1^fl/fl^ mice (open triangle) and FoxP3^GFP/DTR^CD4^Cre/+^Bob1^fl/fl^ mice (closed triangle). Similar results were obtained across two to three independent experiments (**d**–**g**).

We next conducted a comparative transcriptome analysis to better understand the differences between CD4^Cre/+^Bob1^fl/fl^ Tfh cells and control CD4^+/+^Bob1^fl/fl^ Tfh cells. After immunization with NP-CGG, Tfh cells were sorted and used for RNA sequencing (RNAseq) analysis (Fig. 3a). As expected from the results demonstrated in Fig. 1 and Fig. 2, compared to CD4^+/+^Bob1^fl/fl^ Tfh cells, CD4^Cre/+^Bob1^fl/fl^ Tfh cells showed lower expression of *Icos* (Fig. 3b and d). Additionally, the expression level of *Il4* was also significantly reduced in CD4^Cre/+^Bob1^fl/fl^ Tfh cells, whereas the levels of *Bcl6*, *Il21*, *Pou2f1* (Oct-1), and *Pou2f2* (Oct-2) were comparable between CD4^+/+^Bob1^fl/fl^ Tfh and CD4^Cre/+^Bob1^fl/fl^ Tfh cells (Fig. 3b and d). Unexpectedly, inhibitory cell surface receptors such as lymphocyte activating gene 3 (Lag3) and T cell immunoreceptor with Ig and ITIM domains (Tigit) were markedly upregulated in CD4^Cre/+^Bob1^fl/fl^ Tfh cells, as confirmed by flow cytometric analysis (Fig. 3b and e, f). Studies using a normalized enrichment score (NES) based on gene set enrichment analysis (GSEA) suggested that the Bob1 deficiency in Tfh cells led to the induction of gene sets including negative regulation of immune system and T cell activation (Fig. 3c, Supplementary Fig. 7a), which may, at least partly, account for the temporally increased number of CD4^Cre/+^Bob1^fl/fl^ Tfh cells shown in Fig. 2b. GSEA also indicated that Bob1 had a possible capacity to preserve metabolic pathways associated with cellular respiration in Tfh cells (Fig. 3c, Supplementary Fig. 7a). Indeed, physiological examination by a flux analyzer showed the reduced mitochondrial respiratory capacity of CD4^Cre/+^Bob1^fl/fl^ Tfh cells compared to CD4^+/+^Bob1^fl/fl^ Tfh cells (Fig. 3g; refs. ^31,32^). Moreover, RNAseq analysis of CD4^Cre/+^Bob1^fl/fl^ Tfh cells further indicated upregulated levels of sclerostin domain-containing protein 1 (*Sostdc1*), a secretory molecule in Tfh cells, which promotes the differentiation of Tfr cells (Fig. 3b and d; refs. ^33,34^). It was also found that Bob1 in Tfh cells upregulated peptidyl arginine deiminase 4 (*Padi4*), which catalyzes protein citrullination and is associated with the pathogenesis of rheumatoid arthritis (Fig. 3b and d; ref. ^35^). *Padi4*, a Tfh-related gene^36^, was highly expressed in Tfh cells, but not in B cells and other effector T cells, as indicated by the analysis of FoxP3^GFP/DTR^ mice (Supplementary Fig. 7b). The results of the RNAseq study further revealed that myocyte enhancer factor 2b (*Mef2b*) was preferentially expressed in Tfh cells under the control of Bob1 (Fig. 3b and d). Mef2b, which was initially identified as a transcription factor specific to GC B cells, forms a protein complex in collaboration with Bob1 to regulate the locus control region of Bcl6; however, the functional significance of Mef2b in Tfh cells remains unknown^37^. Overall, these findings demonstrate that Bob1 controls cardinal regulatory molecules in Tfh cells, such as cell surface molecules regulating interactions with B cells and essential metabolic pathways. Therefore, Tfh cells can maintain their integrity during humoral responses.

**Fig. 4:**
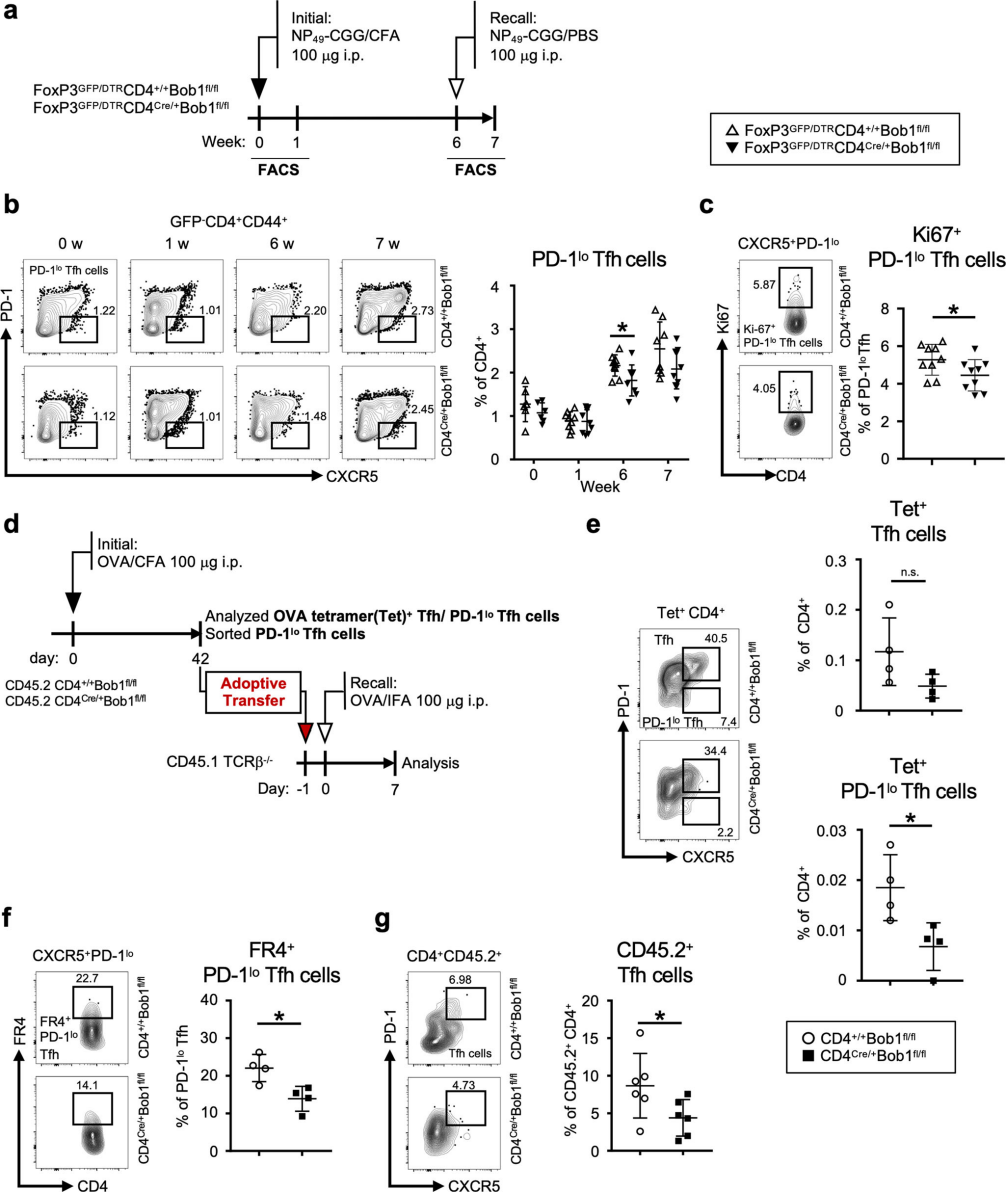
Bob1 supports the memory stage of Tfh cells. **a** Experimental protocol for the immunization of FoxP3^GFP/DTR^CD4^Cre/+^Bob1^fl/fl^ mice and control FoxP3^GFP/DTR^CD4^+/+^Bob1^fl/fl^ mice. NP_49_-CGG was injected intraperitoneally with CFA on day 0 and with PBS on day 42. **b**, **c** Representative flow cytometric profiles and graphs of Tfh cell subsets in spleens. Specimens were analyzed on days 0, 7, 42, and 49 in (**b**), and on day 42 in (**c**). **b** Profiles of PD-1^lo^ Tfh cells (GFP^-^B220^-^CD4^+^CD44^+^CXCR5^+^PD-1^lo^) and graphs showing the % of PD-1^lo^ Tfh cells among the total CD4^+^ T cells. *P = 0.0334. **c** Profiles of Ki67^+^PD-1^lo^ Tfh cells and graphs showing the % of Ki67^+^PD-1^lo^ Tfh cells among the total PD-1^lo^ Tfh cells. *P = 0.0472. **d**–**g** Adoptive cell transfer experiments of PD-1^lo^ Tfh cells using TCRβ^−/−^ mice. **d** Experimental protocol for immunized cell transfer. OVA was administered intraperitoneally with CFA on day 0 to CD45.2^+^CD4^Cre/+^Bob1^fl/fl^ mice and control CD45.2^+^CD4^+/+^Bob1^fl/fl^ mice. PD-1^lo^ Tfh cells (B220^-^CD4^+^CD44^+^CXCR5^+^PD-1^lo^) from the spleen were sorted on day 42 and intravenously transferred into CD45.1^+^TCRβ^−/−^ hosts. The next day, mice were intraperitoneally immunized with OVA in incomplete Freund’s adjuvant (IFA). Seven days later, spleen cells were analyzed. **e**, **f** Flow cytometric analysis of PD-1^lo^ Tfh cells, prior to being injected into CD45.1^+^TCRβ^−/−^ hosts. **e** Representative profiles of OVA-specific tetramer-positive (Tet^+^) Tfh cells and Tet^+^ PD-1^lo^ Tfh cells. Graphs showing the % of Tet^+^ Tfh cells and Tet^+^ PD-1^lo^ Tfh cells among the total CD4^+^ T cells. *P = 0.0286. **f** Representative profiles of FR4^+^PD-1^lo^ Tfh cells and graphs showing the % of FR4^+^PD-1^lo^ Tfh cells among the total PD-1^lo^ Tfh cells. *P = 0.0286. **g** Representative profiles and graphs of CD45.2^+^ Tfh cells in CD45.1^+^TCRβ^−/−^ hosts. The % of CD45.2^+^ Tfh cells among the total CD45.2^+^CD4^+^ T cells are shown. *P = 0.0411. Data represent the mean ± SD of 4–10 mice per group. Statistical significance was analyzed by the Mann-Whitney *U*-test (b-c, e-g); *P < 0.05. Data shown in (**b**, **c**) are from FoxP3^GFP/DTR^CD4^+/+^Bob1^fl/fl^ mice (open triangle) and FoxP3^GFP/DTR^CD4^Cre/+^Bob1^fl/fl^ mice (closed triangle), and data shown in (**e**–**g**) are from CD4^+/+^Bob1^fl/fl^ mice (open circle) and CD4^Cre/+^Bob1^fl/fl^ mice (closed square). Similar results were obtained across two to three independent experiments.

Given the characteristic transcriptomes under the control of Bob1 in Tfh cells, we further examined the role of Bob1 in the fate of Tfh cells. As shown in Fig. 2a, b, similar levels of Tfh cells were observed in both FoxP3^GFP/DTR^CD4^Cre/+^Bob1^fl/fl^ and control FoxP3^GFP/DTR^CD4^+/+^Bob1^fl/fl^ mice at 6 weeks after the initial immunization. Consequently, we focused on CXCR5^+^PD-1^lo^ Tfh cells (PD-1^lo^ Tfh cells), a subset identified in the later phases of the immune response that could be activated for secondary antigen exposure^38,39^. Immunizing control FoxP3^GFP/DTR^CD4^+/+^Bob1^fl/fl^ mice with NP-CGG led to an increase in PD-1^lo^ Tfh cells 6 weeks prior to the second immunization challenge (Fig. 4a, b). This might coincide with the increased level of circulating memory Tfh cells in the peripheral blood of FoxP3^GFP/DTR^CD4^+/+^Bob1^fl/fl^ mice^40–42^. Analysis of FoxP3^GFP/DTR^CD4^Cre/+^Bob1^fl/fl^ mice revealed a reduced level of PD-1^lo^ Tfh cells in comparison to FoxP3^GFP/DTR^CD4^+/+^Bob1^fl/fl^ mice (Fig. 4b). The proliferative potential of PD-1^lo^CD4^Cre/+^Bob1^fl/fl^ Tfh cells appeared to be decreased compared to that of PD-1^lo^CD4^+/+^Bob1^fl/fl^ Tfh cells, as measured by the expression level of Ki67, suggesting that PD-1^lo^CD4^Cre/+^Bob1^fl/fl^ Tfh cells exhibit lower proliferative potential than control PD-1^lo^CD4^+/+^Bob1^fl/fl^ Tfh cells (Fig. 4c). Moreover, when mice were immunized with ovalbumin (OVA), the frequencies of PD-1^lo^ Tfh cells bound to OVA-specific MHC class II tetramer and PD-1^lo^ Tfh cells presenting folate receptor 4 (FR4) as a marker of memory Tfh cells in CD4^Cre/+^Bob1^fl/fl^ mice was lower than those of CD4^+/+^Bob1^fl/fl^ mice (Fig. 4d–f, Supplementary Fig. 8a, b; ref. ^43^). Adoptive cell transfer experiments using Ly5.1-congenic T cell receptor β-chain (TCRβ)-deficient mice (CD45.1) showed that PD-1^lo^ Tfh cells from CD4^Cre/+^Bob1^fl/fl^ mice (CD45.2) had a low potential for activation after the second immunization (Fig. 4d, g). Taken together, these observations suggest that Bob1 likely regulates the recall responses of Tfh cells following their initial encounter with an antigen.

### Bob1 promotes the reactivation of Tfh cells from the memory T pool

**Fig. 5:**
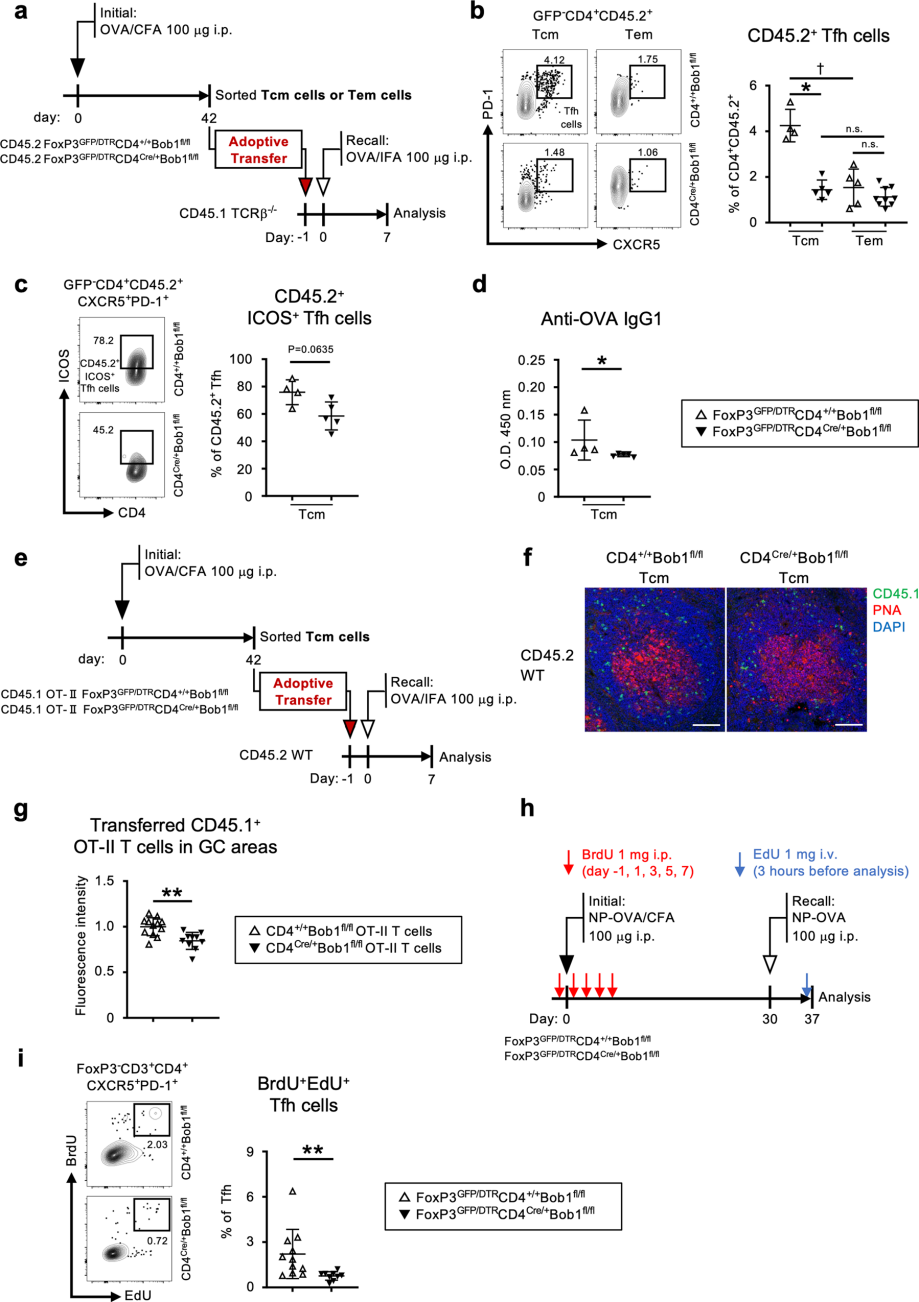
Bob1 regulates the reactivation of Tfh cells from the Tcm pool. **a**–**d** Adoptive cell transfer experiments of CD4^+^ Tcm and Tem cells. **a** Experimental protocol for immunized cell transfer. OVA was administered intraperitoneally with CFA on day 0 to CD45.2^+^FoxP3^GFP/DTR^CD4^Cre/+^Bob1^fl/fl^ mice and control CD45.2^+^FoxP3^GFP/DTR^CD4^+/+^Bob1^fl/fl^ mice. Tcm cells (GFP^-^B220^-^CD4^+^CD44^+^CD62L^+^) or Tem cells (GFP^-^B220^-^CD4^+^CD44^+^CD62L^-^) from the spleens of immunized mice were sorted on day 42 and transferred to CD45.1^+^TCRβ^−/−^ mice via tail veins using the sorting strategy shown in Supplementary Fig. 9. The next day, mice were immunized intraperitoneally with OVA in IFA, and then the sera and spleen cells were analyzed 7 days later. **b** Representative flow cytometric profiles and graphs of CD45.2^+^ Tfh cells (CD4^+^ CD45.2^+^CXCR5^+^PD-1^+^) in CD45.1^+^TCRβ^−/−^ hosts transferred with CD45.2^+^ Tcm or Tem cells. The % of CD45.2^+^ Tfh cells among the total CD4^+^CD45.2^+^ T cells are shown. *P = 0.0159 (CD4^+/+^Bob1^fl/fl^ Tcm cells vs. CD4^Cre/+^Bob1^fl/fl^ Tcm cells) and ^†^*P* = 0.0159 (CD4^+/+^Bob1^fl/fl^ Tcm cells vs. CD4^+/+^Bob1^fl/fl^ Tem cells). **c** Representative flow cytometric profiles and graphs of CD45.2^+^ICOS^+^ Tfh cells in CD45.1^+^TCRβ^−/−^ hosts transferred with CD45.2^+^ Tcm cells. The % of CD45.2^+^ICOS^+^ Tfh cells among the total CD45.2^+^ Tfh cells are shown. **d** Serum levels of OVA-specific IgG1 antibodies in CD45.1^+^TCRβ^−/−^ hosts transferred with CD45.2^+^ Tcm cells as assessed by ELISA. *P = 0.0159. **e**–**g** Histological distribution of Tfh cells originated from CD4^+^ Tcm cells. **e** Experimental protocol for immunized cell transfer. OVA was administered intraperitoneally with CFA on day 0 to CD45.1^+^OT-II^+^FoxP3^GFP/DTR^CD4^Cre/+^Bob1^fl/fl^ mice and control CD45.1^+^OT-II^+^FoxP3^GFP/DTR^CD4^+/+^Bob1^fl/fl^ mice. CD45.1^+^OT-II^+^CD4^+^ Tcm cells were sorted on day 42 and then intravenously transferred into CD45.2^+^ wild-type mice. The next day, mice were immunized intraperitoneally with OVA emulsified in IFA. Seven days later, spleens were examined by immunostaining with a laser confocal microscope. **f** Representative confocal images of spleen sections stained for CD45.1 (green), PNA (red), and DAPI (blue). Scale bar: 50 μm. **g** Graphs show the fluorescence intensity of transferred cells (CD45.1^+^OT-II^+^) localized within the PNA^+^ GC areas of CD45.2^+^ wild-type hosts. Imaging data were analyzed by ZEN software. **P = 0.0020. **h**, **i** Dual DNA labeling experiment. **h** Experimental protocol for immunization in FoxP3^GFP/DTR^CD4^Cre/+^Bob1^fl/fl^ mice and control FoxP3^GFP/DTR^CD4^+/+^Bob1^fl/fl^ mice. NP_16_-OVA was injected intraperitoneally with CFA on day 0 and with PBS on day 30. BrdU was administered intraperitoneally on days −1, 1, 3, 5, and 7. On day 37, EdU was administered through the tail vein 3 h before analysis. **i** Representative flow cytometric profiles and graphs of BrdU^+^EdU^+^ Tfh cells (FoxP3^-^CD3^+^CD4^+^CXCR5^+^PD-1^+^) derived from spleens. The % of BrdU^+^EdU^+^ Tfh cells among the total Tfh cells are shown. **P = 0.0015. Data represent the mean ± SD in (**b**, **c**, **d**, **g**, **i**) of 4–11 mice per group. Statistical significance was analyzed by the Mann-Whitney *U*-test for all groups: *P < 0.05, **P < 0.01. Data in (**b**–**d**) are shown from FoxP3^GFP/DTR^CD4^+/+^Bob1^fl/fl^ mice (open triangle) and FoxP3^GFP/DTR^CD4^Cre/+^Bob1^fl/fl^ mice (closed triangle), and data in (**g**) are shown from CD45.1^+^OT-II^+^FoxP3^GFP/DTR^CD4^+/+^Bob1^fl/fl^ mice (open triangle) and CD45.1^+^OT-II^+^FoxP3^GFP/DTR^CD4^Cre/+^Bob1^fl/fl^ mice (closed triangle). Data in (**i**) are shown from FoxP3^GFP/DTR^CD4^+/+^Bob1^fl/fl^ mice (open triangle) and FoxP3^GFP/DTR^CD4^Cre/+^Bob1^fl/fl^ mice (closed triangle). Similar results were obtained across two to three independent experiments.

To investigate the functional significance of Bob1 in controlling the memory function of Tfh cells, adoptive cell transfer experiments were performed using congenic TCRβ-deficient hosts (CD45.1) that were intravenously injected with central or effector memory CD4^+^ T cells (CD4^+^ Tcm cells or CD4^+^ Tem cells, respectively) derived from immunized CD45.2 mice (Fig. 5a, Supplementary Fig. 9). In this experiment, CD45.2^+^ Tfh cells in the CD45.1^+^ hosts were assumed to be Tfh cells reactivated from the memory CD4^+^ T cell pools after rechallenge with antigen. The results showed an increased level of Tfh cells reactivated from CD4^+^ Tcm cells compared to those reactivated from CD4^+^ Tem cells in CD4^+/+^Bob1^fl/fl^ mice (Fig. 5b). These findings are consistent with those of previous studies indicating that CD4^+^ Tcm cells were a more efficient source of Tfh cells during recall responses compared to CD4^+^ Tem cells^44^. In contrast to the control experiments, Tfh cells were poorly reactivated from CD4^Cre/+^Bob1^fl/fl^ Tcm cells in TCRβ-deficient hosts (Fig. 5b). Interestingly, Tfh cells reactivated from CD4^Cre/+^Bob1^fl/fl^ Tcm cells in TCRβ-deficient mice showed low expression of ICOS (*P* = 0.0635; Fig. 5c). Consistent with these observations, the level of antigen-specific IgG1 in TCRβ-deficient mice was lower when transferred with CD4^Cre/+^Bob1^fl/fl^ Tcm cells compared to CD4^+/+^Bob1^fl/fl^ Tcm cells (Fig. 5d). Moreover, reactivated Tfh cells from OT-II^+^CD4^Cre/+^Bob1^fl/fl^ Tcm cells exhibited reduced localization to GCs compared to OT-II^+^CD4^+/+^Bob1^fl/fl^ Tcm cells, as assessed using the OT-II system, which provided transfer of CD4^+^ Tcm cells into wild-type mice (Fig. 5e–g). This suggests that Bob1 has a critical role in retrieving Tfh functions from the Tcm pool to shape specific humoral responses. Based on these results, we examined Tfh cells using the dual-pulse method for DNA labeling^45–47^. To trace cell division induced by the multiple stimuli of antigens, the two thymidine analogs, 5-bromo-2’-deoxyuridine (BrdU) and 5-ethynyl-2´-deoxyuridine (EdU), were administered around the first and second immunizations with a 30-day interval, respectively (Fig. 5h). The Tfh cell population incorporating BrdU and EdU (BrdU^+^EdU^+^ Tfh cells) was considered to include Tfh cells reactivated from memory CD4^+^ T cells. The results demonstrated that the level of BrdU^+^EdU^+^CD4^Cre/+^Bob1^fl/fl^ Tfh cells was almost half that of the control BrdU^+^EdU^+^CD4^+/+^Bob1^fl/fl^ Tfh cells (Fig. 5i). Taken together, these results suggest that Bob1 plays a role in efficiently recalling Tfh cells from the Tcm pool.

### Bob1-expressing CD4^+^ T cells induce allergic inflammation

**Fig. 6:**
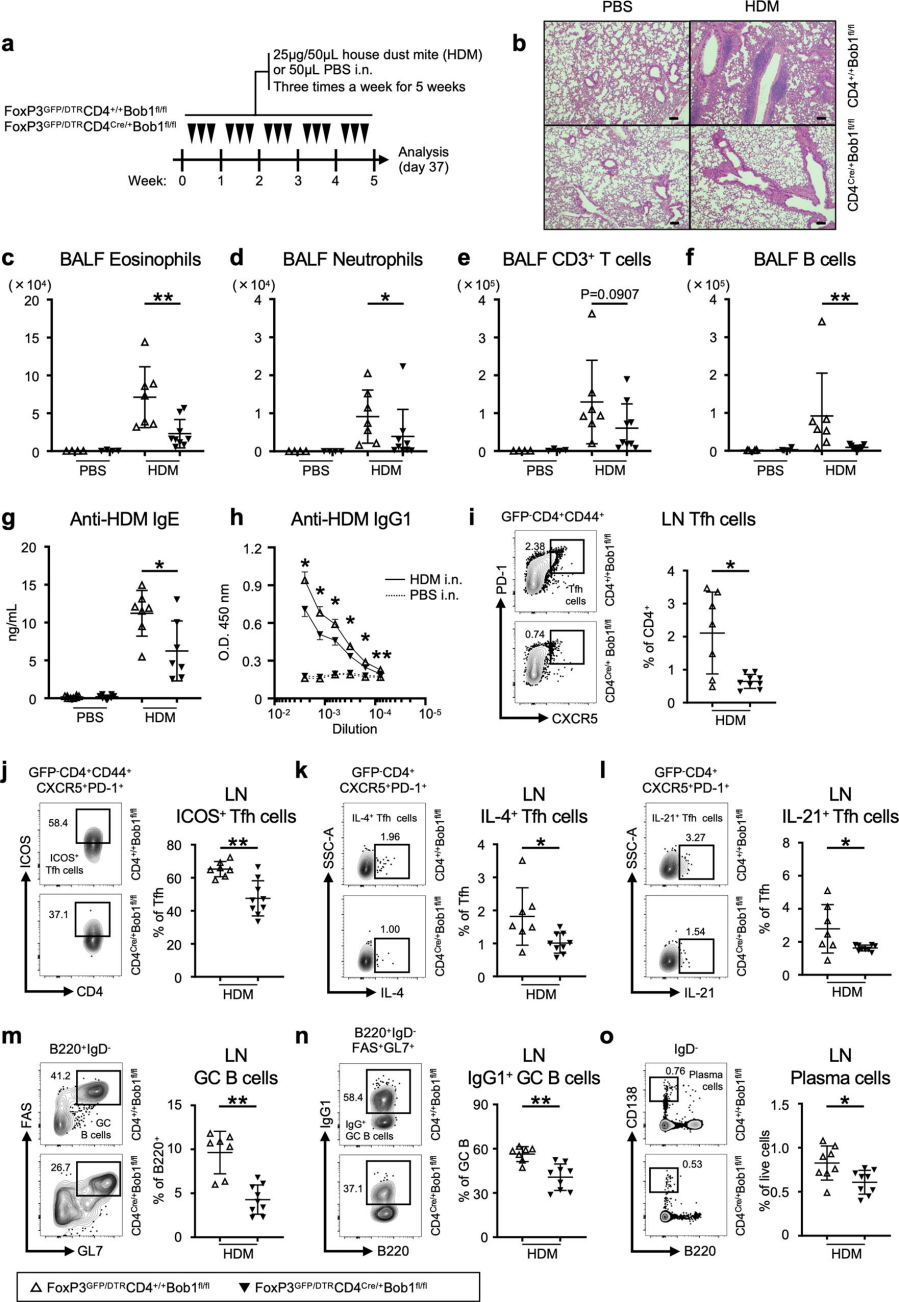
Analysis of HDM-induced allergic asthma models. **a** Experimental protocol for HDM exposure to FoxP3^GFP/DTR^CD4^Cre/+^Bob1^fl/fl^ mice and control FoxP3^GFP/DTR^CD4^+/+^Bob1^fl/fl^ mice. Mice were sensitized by the intranasal instillation of 25 μg HDM in 50 μL saline (PBS) or 50 μL saline alone as a control for 3 days per week consecutively for 5 weeks, before analysis on day 37. **b** Representative microscopy images of formalin-fixed paraffin-embedded tissue sections of pulmonary lesions on day 37 stained with hematoxylin and eosin. Scale bar: 100 μm. **c**–**f** Number of inflammatory and immune cells in the BALF on day 37 assessed by flow cytometry following the gating strategies shown in Supplementary Fig. 10. **c** Eosinophils (CD3^-^Gr-1^-^CD11b^+^CD11c^-^), **P = 0.0052. **d** Neutrophils (CD3^-^Gr-1^+^CD11b^+^CD11c^-^), *P = 0.0418. **e** CD3^+^ T cells (CD3^+^CD11c^-^MHCII^-^). **f** B cells (B220^+^CD3^-^Gr-1^-^CD11b^-^CD11c^-^MHC-II^+^), **P = 0.0033. **g**, **h** Serum levels of HDM-specific antibodies on day 37 assessed by ELISA. **g** Serum levels of HDM-specific IgE, *P = 0.0379. **h** Serum levels of HDM-specific IgG1 in different dilutions are indicated by solid lines. The levels were compared between FoxP3^GFP/DTR^CD4^+/+^Bob1^fl/fl^ and FoxP3^GFP/DTR^CD4^Cre/+^Bob1^fl/fl^ mice with statistics as indicated. *P = 0.0256, *P = 0.0256, *P = 0.0304, *P = 0.0455, *P = 0.0221, **P = 0.0030, in order from left to right. **i**–**o** Representative flow cytometric profiles and graphs of lymphocyte subsets within the mediastinal lymph nodes on day 37 assessed by flow cytometry following the gating strategies shown in Supplementary Fig. 5a, b. **i** Tfh cells (GFP^-^B220^-^CD4^+^CD44^+^CXCR5^+^PD-1^+^), *P = 0.0283. **j** ICOS^+^ Tfh cells, **P = 0.0052. **k** IL-4^+^ Tfh cells, *P = 0.0164. **l** IL-21^+^ Tfh cells, *P = 0.0287. **m** GC B cells (B220^+^IgD^-^FAS^+^GL7^+^), **P = 0.0012. **n** IgG1^+^ GC B cells, **P = 0.0012. **o** Plasma cells (B220^lo^IgD^-^CD138^hi^), *P = 0.0222. Data represent the mean ± SD (**c**–**g**, **i**–**o**) or the mean ± SEM (**h**) of 4–9 mice per group. Statistical significance was analyzed by the Mann–Whitney *U*-test; *P < 0.05, **P < 0.01, ***P < 0.001. Data in (**c**–**o**) are shown for FoxP3^GFP/DTR^CD4^+/+^Bob1^fl/fl^ mice (open triangle) and FoxP3^GFP/DTR^CD4^Cre/+^Bob1^fl/fl^ mice (closed triangle). Similar results were obtained across three independent experiments. See also Supplementary Fig. 11.

Finally, we investigated the pathological role of Bob1^+^ Tfh cells in an experimental HDM-induced bronchial asthma model, in which intranasal administration of HDM for 5 weeks leads to chronic airway inflammation resembling clinical allergic asthma (Fig. 6a; ref. ^27^). Histopathological examination demonstrated the massive infiltration of inflammatory cells in the lung tissues of control FoxP3^GFP/DTR^CD4^+/+^Bob1^fl/fl^ mice (Fig. 6b). Conversely, FoxP3^GFP/DTR^CD4^Cre/+^Bob1^fl/fl^ mice exhibited lower severity of pulmonary inflammation and fewer inflammatory cell infiltrates compared to the controls. Flow cytometric analysis showed that the bronchoalveolar lavage fluid (BALF) of FoxP3^GFP/DTR^CD4^Cre/+^Bob1^fl/fl^ mice had lower levels of granulocytes including eosinophils and neutrophils, and T and B lymphocytes than the BALF of control mice (Fig. 6c–f, Supplementary Fig. 10). Accordingly, goblet cell hyperplasia in the airways was observed in FoxP3^GFP/DTR^CD4^Cre/+^Bob1^fl/fl^ mice to a lesser extent than in the controls (Supplementary Fig. 11a, b). The levels of macrophages and dendritic cells in the BALF were comparable between FoxP3^GFP/DTR^CD4^Cre/+^Bob1^fl/fl^ and the control mice (Supplementary Fig. 11c, d). Remarkably, the serum levels of anti-HDM IgE and IgG1 antibodies were markedly decreased in FoxP3^GFP/DTR^CD4^Cre/+^Bob1^fl/fl^ mice, suggesting their poor ability to produce HDM-specific antibodies (Fig. 6g, h). Consistent with these results, the proportions of Tfh cells in the mediastinal (draining) lymph nodes were significantly reduced in FoxP3^GFP/DTR^CD4^Cre/+^Bob1^fl/fl^ mice compared to those in the controls  (Fig. 6i, Supplementary Fig. 5a). Among the Tfh cells in the lymph nodes, the level of IL-13-expressing Tfh cells (Tfh13 cells) in FoxP3^GFP/DTR^CD4^Cre/+^Bob1^fl/fl^ mice was similar to that in the controls (Supplementary Fig. 11e; ref. ^48^). Further analysis demonstrated the lower levels of ICOS, IL-4, and IL-21 in Tfh cells from FoxP3^GFP/DTR^CD4^Cre/+^Bob1^fl/fl^ mice compared to those in Tfh cells from the controls (Fig. 6j–l). In the mediastinal lymph nodes, GC-related B-cell populations, including IgG1^+^ GC B cells and plasma cells, remained lower in FoxP3^GFP/DTR^CD4^Cre/+^Bob1^fl/fl^ mice than those in the controls (Fig. 6m–o, Supplementary Fig. 5b). The levels of IL-4, IL-13, and IL-21 in non-Tfh cells within the mediastinal lymph nodes of FoxP3^GFP/DTR^CD4^Cre/+^Bob1^fl/fl^ mice were not significantly different from those in the controls (Supplementary Fig. 11f–h). Collectively, these results suggest that Bob1 promotes Tfh cells for pathological immune conditions during the repetitive inhalation of HDM.

## Discussion

In this study, we report the role of a Bob1 coactivator in the regulation of Tfh cells to maintain the durable generation of antigen-specific antibodies. Studies using T-dependent antigens have suggested the requirement of Bob1 for the reactivation and memory formation of Tfh cells upon multiple antigen challenges. Studies on the allergic asthma models further indicated the role of Bob1 in Tfh cell regulation, whereby HDM-specific IgE and IgG antibodies are steadily produced. As Bcl6^+^CD4^+^ T cells can promote antigen-specific IgE responses, it has been suggested that Tfh cells are actively involved in allergic conditions^48–50^. The defective humoral immune responses in the conditional Bob1 deficient mice are probably, at least in part, attributable to the downregulation of ICOS in Tfh cells, given the functional significance of ICOS in Tfh cells for the development of plasma cells, the generation of class-switched antibodies, and the maintenance of a Tcm pool^29,30,51,52^. In terms of respiratory capacity, CCR7^hi^CD137^lo^ memory precursor CD4^+^ T cells demonstrate a higher respiratory capacity compared to CCR7^lo^CD137^hi^ effector CD4^+^ T cells and further exhibit a greater propensity for subsequent memory formation^53^. It was found that Bob1-dependent mechanisms in Tfh cells involve the cellular respiration pathway associated with the metabolism of intrinsic NAD and ATP, which is probably required to support memory Tfh cells^31,38,43,53–55^. IL-4 derived from Tfh cells, rather than Th2 cells, has been proposed to play a key role in IgE generation^48,56^. Thus, the reduced levels of antigen-specific IgE and IgG in the conditional Bob1 deficient mice are considered to be partly caused by their poor ability to present IL-4. In this context, Bob1 is postulated as a vital coactivator for the programming of Tfh cells to continuously produce high-affinity class-switched antibodies. Considering that circulating Tfh cells provide rapid and robust B-cell help following secondary antigen exposure^57,58^, Bob1 in Tfh cells might play an essential role in maintaining the integrity of systemic humoral immunity.

Based on our current understanding of the effector subsets of CD4^+^ T cells, the pathologic basis of allergy may be associated with the functional dichotomy of Th2 cells, involved in the characteristic tissue inflammation, and Tfh cells, involved in IgE production, both hallmark features of allergic states^4–7,59^. Upon exposure to HDM administered via the airway, the conditional Bob1 deficient mice exhibited a diminished population of Tfh cells present in the regional lymph nodes and decreased inflammation of the lung tissues. In vitro studies demonstrated that Bob1 can control PU.1 promoter activity, thereby regulating Th2 cytokine production^15^. However, there was no significant change in Th2 cytokine production by non-Tfh cells in the HDM-induced asthma models of the conditional Bob1 deficient mice. Of note, Tfh cells have the ability to convert into Th2 cells within the tissue lesions of HDM-induced asthma^60^. In this context, Tfh cells in the lung lesions of the conditional Bob1 deficient mice may not fully promote Th2-skewed airway inflammation under the repetitive inhalation of HDM. Although the regulatory mechanism related to the plasticity of Tfh cells remains controversial, Tfh cells in the memory phase may exhibit high multipotency towards Th2 polarization following recall responses under allergic conditions^7^. Generally, in response to repeated antigen exposure, primed memory T cells are rapidly activated and maintained for extended periods. Experimental evidence indicating that Bob1 regulates the activation of Tfh cells from the central memory CD4^+^ T-cell pool implies the involvement of Bob1 in the dynamics of primed memory Tfh cells. Bob1 acts through binding to Oct family transcription factors, which set programs related to cell function by direct access to DNA and chromatin in different configurations^61^. Oct-1 and Oct-2 take part in the dynamic regulation of long-range cis-regulatory elements such as a locus control region or a super-enhancer^62,63^. Although the functional specificity of Oct-1 and Oct-2 in CD4^+^ T cell subsets is not fully understood, Bob1 promotes critical epigenetic changes during the formation of memory CD4^+^ T cells by its binding to Oct-1 and Jmjd1a of a histone lysine demethylase, which may be accompanied by the expression of ICOS^64^. Notably, the memory CD4^+^ T cell compartment contains long-lived Tfh cells, which have broad recall capacity after multiple encounters with antigens^43^. Moreover, memory CD4^+^ T cells are believed to play a crucial role in promoting allergic responses, and their presence is necessary for the development of allergic airway inflammation in response to allergens^65^. Subsequently, the Bob1-dependent phase of Tfh cells may support the intrinsic function and the potential for plasticity, ensuring their primary role, as well as the secondary responses. In addition to our study, it will be important to conduct further investigation into the enduring maintenance mechanisms of CXCR5^+^PD-1^+^ Tfh cells as a memory phenotype, facilitated by Bob1^43^.

Bcl6, a transcription factor that defines the lineage of Tfh cells, plays a cardinal role in establishing the identity of Tfh cells by multiple distinct mechanisms via a broad range of promoters^66,67^. Our study showed that Tfh cells highly express Bob1 and Oct-2, similar to B cells in the GCs. In B cells, Bob1, Mef2b, and Oct-2 form a ternary complex that regulates the locus control region on the Bcl6 promoter^68^. Although Bob1-deficient Tfh cells show downregulated Mef2b, we failed to find a significant difference in the expression levels of Bcl6 between Bob1-deficient and control Tfh cells, suggesting that the mechanism underlying Bcl6 expression in Tfh cells is different from that in B cells. The deficiency of Bcl6 in CD4^+^ T cells can elicit excessive effector functions in Th2 cells^50^. Meanwhile, the loss of Bob1 in CD4^+^ T cells did not significantly hamper the initial humoral responses, and comparable levels of Bcl6 expression were observed in Bob1-deficient and control Tfh cells. Therefore, the function of Bob1 on the Bcl6-Blimp1 axis during the process of CD4^+^ T cell differentiation is likely limited. The loss of Bob1 in Tfh cells induced the upregulation of Tfr cells, which would influence Tfh cell function during B cell activation^5^. Although the precise mechanism by which Bob1 influences the regulation of Tfr cells in immune settings was not fully explored in this study, the elevated expression of Sostdc1 in the Bob1-deficient Tfh cells could be regarded as a potential mechanism for the augmented population of Tfr cells, as Sostdc1 has been shown to promote their development^34^.

In summary, we present evidence that Bob1 plays a crucial role in orchestrating the transcriptional program that regulates Tfh cells, facilitating long-term humoral protection against foreign antigens. Additionally, our findings suggest the involvement of Bob1 in allergic inflammation. On this basis, targeting Bob1 in allergen-specific Tfh cells could represent a viable approach for treating allergic conditions. Future studies exploring the unique regulation of primary and memory Tfh cells have the potential to yield novel insights into strategies to modulate immune responses in both protective and pathological contexts.

## Methods

### Clinical specimens

Tonsil samples for the analysis of lymphocytes were obtained from tonsillectomy cases with tonsillar hypertrophy or recurrent tonsillitis at Sapporo Medical University Hospital. The tissues were carefully cut and gently mashed to extract all lymphocytes for further analysis. The protocol for collecting and analyzing patient samples was ethically approved by the Institutional Review Board of Sapporo Medical University Hospital, Japan (IRB#25-39, IRB#292-83). All participants provided written informed consent in accordance with the Declaration of Helsinki. All ethical regulations relevant to human research participants were followed.

### Mice and immunization

Bob1^fl/+^ mice on a C57BL/6 background were established as summarized in Supplementary Fig. 1 (Accession No. CDB0030E: https://large.riken.jp/distribution/mutant-list.html). Bob1^fl/+^ mice were designed to have a specific deletion of the entire *Pou2af1* gene using the Cre/loxP system by CRISPR/Cas9 genome editing technology. CD4^Cre/+^ (B6.Cg-Tg(CD4-Cre)1Cwi/BfluJ), FoxP3^GFP/DTR^ (B6.129Cg-Foxp3^tm3(DTR/GFP)Ayr^/J), OT-II (B6.Cg-Tg(TcraTcrb)425Cbn/J), TCRβ-deficient (TCRβ^−/−^, B6.129P2-Tcrb^tm1Mom^/J), and Bob1-deficient (Bob1^−/−^, B6;129-Pou2af1^tm1Rgr^/J) mice were obtained from the Jackson Laboratory (Bar Harbor, ME). Ly5.1-congenic C57BL/6 mice (CD45.1, B6.SJL-Ptprc^a^/Rbrc) and C57BL/6 wild-type (WT) mice (CD45.2) were obtained from RIKEN Bioresource Center (Tsukuba, Japan) and Sankyo Laboratory (Tokyo, Japan), respectively. For the in vivo depletion of FoxP3^+^ cells, FoxP3^GFP/DTR^ mice were intraperitoneally administered 500 ng of diphtheria toxin (Fujifilm Wako Pure Chemical, Tokyo, Japan) in 200 μL PBS. Mice were kept under specific pathogen-free conditions in the animal facility of Sapporo Medical University. Age- (6–12 weeks) and sex-matched littermate mice in each group were analyzed, unless otherwise stated. For immunization, mice were intraperitoneally administered the following immunogens: 100 μg of NP_49_-CGG (Biosearch Technologies, Radnor, PA), 100 μg of NP_16_-OVA (Biosearch Technologies), 100 μg of OVA (Sigma-Aldrich, St. Louis, MO), or 200 μL of 20% (v/v) SRBCs (Japan Bio Serum, Tokyo, Japan). Complete and incomplete Freund’s adjuvant (CFA and IFA, respectively; Fujifilm Wako Pure Chemical) were also used in conjunction with the immunogens. Sera were collected from tail veins and stored at −80 °C until examinations. All experiments involving mice were conducted in accordance with institutional and national animal welfare guidelines, following the approved protocols of Sapporo Medical University (#17-110) and the RIKEN Kobe branch (#H17-04-45) for the care and use of animals.

### HDM-induced bronchial asthma model

For the induction of allergic airway inflammation with HDM, mice were anesthetized with isoflurane and intranasally administered 50 μl of PBS containing 25 μg of HDM extract (Greer Laboratories, Lenoir, NC) three times per week for 5 weeks. Control mice received the same volume of PBS only three times per week for 5 weeks. At the end of the fifth week, mice were euthanized, and the BALF was isolated by infusing and recovering 0.5 ml of PBS into the lungs. After centrifugation of the BALF, cells were separated for subsequent fluorescence-activated cell sorting (FACS) analysis. Additionally, the mediastinal lymph nodes were collected under a stereo microscope to obtain cells for FACS analysis. Lungs were fixed using a 10% buffered neutral formalin solution and embedded in paraffin. Formalin-fixed paraffin-embedded tissue sections of the lungs were stained with hematoxylin and eosin (HE) or Periodic acid-Schiff (PAS), and examined under a light microscope.

### Antibodies and reagents

The following antibodies to surface markers were used: anti-human mAbs including anti-CD3e-FITC (SK7), anti-CD4-APC-Cy7 (RPA-T4), anti-CXCR5-PerCP-Cy5.5 (RF8B2), and anti-PD-1-PE (EH12.1), all purchased from BD Biosciences (Franklin Lakes, NJ); anti-CD45RA-BV510 (HI100) purchased from BioLegend (San Diego, CA); anti-mouse mAbs including anti-CD3-PE-Cy7 (145-2C11), anti-CD4-BV510 (RM4-5), anti-CD8-PE (53-6.7), anti-CD8-BV510 (53-6.7), anti-CD11b-BB515 (M1/70), anti-CD11c-PE (N418), anti-CD24-biotin (M1/69), anti-CD43-PE (S7), anti-CD45.2-PE-Cy7 (104), anti-B220-PerCP-Cy5.5 (RA3-6B2), anti-B220-APC (RA3-6B2), anti-CD62L-APC-Cy7 (MEL-14), anti-FAS-PE-Cy7 (Jo2), anti-Siglec-F-BV421 (E50-2440), anti-ICOS-BV421 (7E.17G9), anti-IgM-PE-Cy7 (R6-60.2), anti-IgD-FITC (11-26c.2a), anti-IgG1-FITC (A85-1), anti-Gr-1-APC (RB6-8C5), anti-GATA3-PE (L50-823), anti-RORγt-BV421 (Q31-378), and streptavidin-APC-Cy7, all purchased from BD Biosciences; anti-CD4-APC-Cy7 (GK1.5), anti-CD38-APC (90), anti-CD44-PE-Cy7 (IM7), anti-CD45.1-APC-Cy7 (A20), anti-CD138-APC-Cy7 (281-2), anti-CXCR5-PE (L138D7), anti-CXCR5-APC-Cy7 (L138D7), anti-CXCR5-BV421 (L138D7), anti-LAG3-APC (C9B7W), anti-ICOS-PE (7E.17G9), anti-PD-1-APC (29F.1A12), anti-PD-1-PE-Cy7 (29F.1A12), anti-GL7-PacificBlue (GL7), anti-I-A/I-E-PE-Cy7 (M5/114.15.2), anti-Tbet-APC (4B10), anti-IL-4-BV421 (11B11), and anti-FR4-APC-Fire750 (12A5), all purchased from BioLegend; anti-TIGIT-AF647 (GIGD7), anti-Ki67-eFluor450 (SolA15), anti-FoxP3-FITC (FJK-16s), anti-IL-13-PE-Cy7 (eBio13A), and anti-IL-21-PE (FFA21), all purchased from eBioscience (San Diego, CA). NP conjugated with PE was obtained from Biosearch Technologies. For MHC class II tetramer staining analysis, single-cell suspensions were stained with T-Select I-A^b^ OVA_323–339_ Tetramer-PE (MBL, Tokyo, Japan) in PBS for 1 h at room temperature prior to surface marker staining.

### Flow-cytometric analysis and cell sorting

Single-cell suspensions from tissues were prepared using a cell strainer (70-µm pore size; Corning, New York, NY) and density-gradient centrifugation with Lympholyte (Cedarlane, Burlington, Canada). To analyze the mouse cells, cells were pretreated with an anti-CD16/CD32 mAb (2.4G2; BD Biosciences). Cells were stained with fluorochrome-conjugated monoclonal antibodies in FACS buffer (PBS containing 0.5% BSA and 2 mM EDTA) on ice for 1 h, and cells were analyzed and/or sorted using FACS Canto I or FACS Aria II and III (BD Biosciences) in combination with a Magnisort mouse CD4^+^ T-cell enrichment kit (Thermo Fisher Scientific, Waltham, MA). In all analyses, 7-AAD was used to exclude non-viable cells and discriminate between doublets or multiplets, and samples were analyzed for singlet events with doublet discrimination. The flow cytometry data were analyzed using FACS DiVA and FlowJo software (BD Biosciences). The purity of FACS-sorted cells reached 95% after validation by reanalysis using FACS Canto I. For intracellular protein staining, cells were fixed with transcription factor staining buffer and permeabilization buffer according to the manufacturer’s instructions (Thermo Fisher Scientific) to detect transcription factors. Rabbit anti-Bob1 pAb (C20; Santa Cruz Biotechnology, Santa Cruz, CA) and BV421-conjugated anti-rabbit IgG (poly4064; BioLegend) were used to detect Bob1 in cells. For cytokine staining, cells were plated in RPMI-1640 (Fujifilm Wako Pure Chemical) containing 10% heat-inactivated fetal calf serum (FCS), 100 units ml^−1^ penicillin, 50 μg ml^−1^ streptomycin, 55 μM 2-mercaptoethanol, and 10 mM Hepes (pH 7.4) supplemented with 500 ng ml^−1^ PMA, 1 μg ml^−1^ ionomycin, and 1:1500 GolgiStop (BD Biosciences) and incubated at 37 °C in a humidified atmosphere with 5% CO_2_ for 4 h. After incubation, the cells were stained with antibodies against surface makers and 7-AAD, and fixed with IC fixation buffer according to the manufacturer’s instructions (Thermo Fisher Scientific).

### Enzyme-linked immunosorbent assay (ELISA)

To measure anti-NP or anti-SRBC immunoglobulin titers, a 96-well microtiter plate for ELISA (Corning) was coated with 10 µg ml^−1^ NP_27_-BSA (Biosearch Technologies) or extracts of SRBC in a carbonate-bicarbonate BupH buffer (Thermo Fisher Scientific). An immunogen-coated microtiter plate was treated with 1% BSA in PBS to prevent non-specific background staining and then washed with PBS washing buffer containing 0.02% Tween-20 (Nacalai Tesque, Kyoto, Japan). Properly diluted serum was incubated at room temperature for 2 h. HRP-conjugated IgM, IgG1, IgG2a, IgG2b, and IgG3 antibodies (SouthernBiotech, Birmingham, AL) were incubated separately on plates at room temperature for 2 h. TMB microwell peroxidase substrate (Sera Care, Milford, MA) and 0.2 N H_2_SO_4_ as a stop buffer were used for colorimetric detection. The OD value was measured as the absorbance at 450 nm using a microplate reader (Multiskan FC, Thermo Fisher Scientific). HDM-specific IgG1 was detected using a high-binding plate coated with 5 μg ml^−1^ HDM (Greer Laboratories). HDM-specific IgE in sera was analyzed by an ELISA kit for anti-HDM IgE antibodies in mouse serum according to the manufacturer’s protocols (Chondrex, Woodinville, WA).

### Immunoblotting analysis

Cells were harvested and lysed with CST lysis buffer (Cell Signaling Technology, Danvers, MA) supplemented with a protease inhibitor cocktail (Thermo Fisher Scientific) and subjected to SDS-PAGE. Primary antibodies used for immunoblotting were rat anti-mouse Bob1 mAb (6F10; Santa Cruz Biotechnology) and mouse anti-β-actin mAb (AC-15; Sigma-Aldrich). After incubation with HRP-conjugated secondary antibodies (Thermo Fisher Scientific), the target bands were visualized on the blots using an ECL prime kit (Cytiva, Marlborough, MA) and an imaging analyzer (LuminoGraph; ATTO, Tokyo, Japan).

### Cell cultures

To activate CD4^+^ T cells, 5 × 10^5^ naive CD4^+^ T cells (B220^-^CD4^+^CD44^-^CD62L^+^) were cultured in RPMI-1640 medium (Fujifilm Wako Pure Chemical) containing 10% heat-inactivated FCS, 100 units ml^−1^ penicillin, 50 µg ml^−1^ streptomycin, 55 µM 2-mercaptoethanol, and 10 mM Hepes (pH 7.4) supplemented with 1.5 µg ml^−1^ anti-CD28 antibody (37.51; BioLegend). The cells were incubated for 2 days in a 24-well plate coated with anti-CD3 antibody (10 µg ml^−1^, 145-2C11; BD Biosciences), at 37 °C in a humidified atmosphere of 5% CO_2_. To induce T cell differentiation into Th subsets, 2 × 10^5^ naive CD4^+^ T cells were cultured in the same medium in a 96-well flat-bottom plate coated with anti-CD3 antibody (10 µg ml^−1^, 145-2C11; BioLegend), before adding the following differentiation cocktails: for Th1 cells, 5 µg ml^−1^ anti-IL-4 mAb (11B11; BioLegend) and 10 ng ml^−1^ mouse IL-12p70 (R&D Systems, Minneapolis, MN); for Th2 cells, 5 µg ml^−1^ anti-IFN-γ mAb (R4-6A2; Thermo Fisher Scientific) and 10 ng ml^−1^ mouse IL-4 (PeproTech, Cranbury, NJ); and for Th17 cells, 5 μg ml^−1^ anti-IFN-γ mAb (R4-6A2), 5 μg ml^−1^ anti-IL-4 (11B11), 20 ng ml^−1^ human IL-6 (PeproTech), and 1 ng ml^−1^ human TGF-β1 (R&D Systems). Thereafter, cells were incubated at 37 °C in a humidified atmosphere with 5% CO_2_ for 5 days and the expression levels of Tbet, GATA3, and RORγt were measured by intracellular flow cytometry.

### RNA sequencing analysis

Total RNA was extracted from sorted Tfh cells using TRIzol. RNA sequencing libraries were prepared with a SMART-Seq v4 Ultra Low Input RNA kit and a Nextra XT library preparation kit (Illumina, San Diego, CA) and then sequenced using an Illumina NovaSeq 6000 high-throughput sequencing system (RIKEN Genesis, Tokyo, Japan). Data were analyzed by bioinformatics software (Cutadapt version 1.2.1, PRINSEQ version 0.19.2, TopHat version 2.0.13, Cufflinks version 2.2.1; RIKEN Genesis), and ggVolcanoR software (version 1.0; Monash University, Melbourne, Australia). Differentially expressed genes were assessed with an FDR-adjusted *P*-value < 0.05 between two of the populations, and the fragments per kilobase of transcript per million mapped reads were summed across isoforms to obtain data. Data were analyzed using GraphPad Prism software for heat mapping analysis (version 7.0; GraphPad, San Diego, CA) and GSEA software for GSEA (version 4.1.0; UC San Diego, La Jolla, CA).

### RT-qPCR analysis

Single-strand cDNA was synthesized from total RNA using a High-Capacity cDNA Reverse Transcription kit (Thermo Fisher Scientific). Quantitative PCR analysis was conducted to detect gene-specific products using TaqMan probes with the Light Cycler 96 System (Roche, Basel, Switzerland). The following TaqMan probes were provided by Thermo Fisher Scientific: *Pou2af1* (FAM-MGB, Mm00448326_m1), *Pou2f1* (FAM-MGB, Mm00448332_m1), *Pou2f2* (FAM-MGB, Mm00448353_m1), *Bcl6* (FAM-MGB, Mm00477633_m1), *Icos* (FAM-MGB, Mm00497600_m1), *Il4* (FAM-MGB, Mm00445259_m1), *Il21* (FAM-MGB, Mm00517640_m1), *Sostdc1* (FAM-MGB, Mm03024258_s1), *Padi4* (FAM-MGB, Mm01341658_m1), *Mef2b* (FAM-MGB, Mm00484956_g1), and *Gapdh* (VIC-MGB, Mm99999915_g1) as a control.

### Extracellular flux assay

For the extracellular flux assay, 2 × 10^5^ cells per well were plated into a Seahorse XFe96 cell culture microplate (Agilent Technologies, Santa Clara, CA) with 180 µL of Seahorse XF DMEM assay medium containing 5.5 mM glucose, 1.0 mM sodium pyruvate, 2.0 mM glutamine, and 5.0 mM HEPES (pH 7.4). The oxygen consumption rate (OCR) was measured on a Seahorse XFe96 Bioanalyzer (Agilent Technologies) under a 3-min mixing and 3-min measuring protocol. The assay involved sequential injection of oligomycin (final concentration: 1.0 μM), carbonyl cyanide p-trifluoromethoxyphenylhydrazone (FCCP, final concentration: 2.0 μM), and rote-none/antimycin A mixture (final concentration: 1.0 μM). The OCR values were normalized to the total protein per well after completing the assay.

### Adoptive cell transfer analysis

CD4^+^ T-cell populations (PD-1^lo^ Tfh cells, Tcm cells, or Tem cells) were isolated from the spleens of immunized mice using a Magnisort CD4^+^ T-cell enrichment kit (Thermo Fisher Scientific) and a cell sorter (BD Biosciences). Then, 3 × 10^5^ cells of each CD4^+^ T-cell population were suspended in 200 μl of PBS and transferred into a congenic mouse through tail vein injection. The next day, mice were immunized with an immunogen.

### Laser confocal microscopy

Frozen tissue sections of the spleen were fixed in ice-cold acetone and subjected to immunostaining with Alexa Fluor 488-conjugated anti-mouse CD45.1 mAb (A20; BioLegend) in a moisture chamber at 4 °C overnight. After washing with PBS three times, tissue sections were reacted with Alexa Fluor 594-conjugated lectin PNA (Thermo Fisher Scientific) for 2 h. Following another wash with PBS, tissue sections were stained with 4’,6-diamidino-2-phenylindole (DAPI; Thermo Fisher Scientific) for 10 min to visualize the nuclei. After washing with PBS, the tissue slides were analyzed using an ELYRAS.1 LSM780 laser confocal microscope with ZEN image examiner software (Carl Zeiss, Jena, Germany).

### DNA labeling using thymidine analogs

For in vivo DNA labeling, mice were intraperitoneally administered 1 mg of BrdU and intravenously injected 1 mg of EdU (Sigma-Aldrich) in PBS. To detect incorporated BrdU and EdU in DNA, cells were analyzed with a BrdU assay kit for flow cytometry using an APC-conjugated anti-BrdU antibody (BD Biosciences) and a Click-iT EdU Pacific Blue flow cytometry assay kit utilizing click chemistry of azide-alkyne reactions (Thermo Fisher Scientific). The procedures followed the manufacturer’s protocols.

### Statistics and reproducibility

The Mann-Whitney *U-*test was used to analyze the difference between the two groups. For comparisons of multiple groups, one-way ANOVA with Tukey’s multiple comparison test was used. The sample size and number of replicates were chosen empirically or based on preliminary data to ensure a sufficient level of statistical power for detecting indicated biological effects. All statistical tests were performed with GraphPad Prism (GraphPad Software). *P-*values < 0.05 were considered to show statistically significant differences between groups and are as denoted by asterisks on graphs.

### Reporting summary

Further information on research design is available in the Nature Portfolio Reporting Summary linked to this article.

### Supplementary information

Supplementary Information

Description of Additional Supplementary Files

Supplementary Data

Reporting Summary

## Data Availability

RNA sequencing data generated in this manuscript are available from the Gene Expression Omnibus repository hosted by National Center for Biotechnology Information (Accession number GSE223725). The numerical source data for the graphs in the main and supplementary figures can be found in Supplementary Data.
