# Supplementary material for: Bob1 maintains T follicular helper cells for long-term humoral immunity
Source: Commun Biol. 2024 Feb 15;7:185. doi: 10.1038/s42003-024-05827-0 (PMC10869348; doi:10.1038/s42003-024-05827-0)
Supplement: Supplementary file 1 — Supplementary Information [file 42003_2024_5827_MOESM1_ESM.pdf]

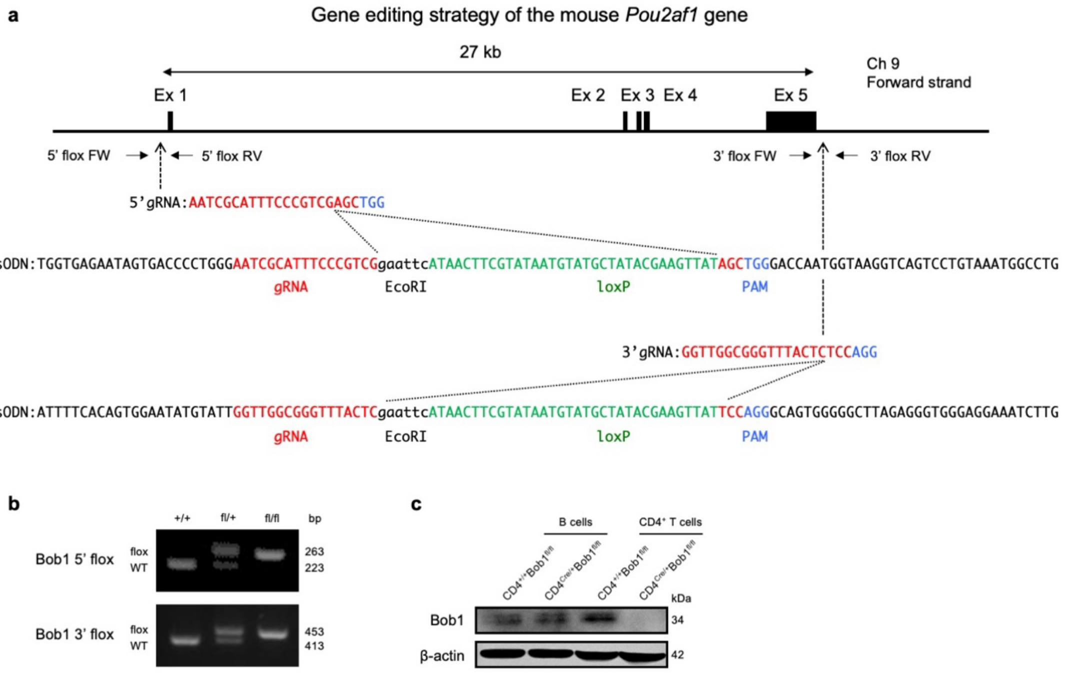

Supplementary Fig. 1. Strategy to establish Bob1$^{fl/fl}$ mice allowing deletion of the entire region of the *Pou2af1* gene

(a) Bob1$^{fl/+}$ mice were generated by a single-strand-oligodeoxynucleotides (ssODNs) mediated knock-in methodology with the CRISPR/Cas9 system using zygotes from wild-type C57BL/6N mice, in which loxP sites flank the entire region of the *Pou2af1* gene encoding Bob1. The gRNAs were designed according to on-target and off-target scoring using the Zhang laboratory website (*Nat Biotechnol.* 31; 827-832, 2013). For electroporation, the zygotes were suspended in Opti-MEM I (Thermo Fisher Scientific) containing two crRNAs (25 ng μl^{-1}), tracrRNA (100 ng μl^{-1}), two ssODNs consisting of a loxP site and an *Eco*RI restriction enzyme recognition site (400 ng μl^{-1}), and Cas9 protein (250 ng μl^{-1}; Thermo Fisher Scientific). The crRNA and tracrRNA were as follows; 5'-crRNA(5'-AAU CGC AUU UCC CGT CGA GCg uuu uag agc uau gcu guu uug-3'), 3'-crRNA(5'-GGU UGG CGG GUU UAC UCU CCg uuu uag agc uau gcu guu uug-3'), and tracrRNA (5'-AAA CAG CAU AGC AAG UUA AAA UAA GGC UAG UCC GUU AUC AAC UUG AAA AAG UGG CAC CGA GUC GGU GCU-3') (FASMAC, Atsugi, Kanagawa, Japan). The ssODN sequences are indicated. CUY21 EDIT II and LF501PT1-10 platinum plate electro code (BEX, Tokyo, Japan) were used for electroporation under conditions of 30 V (3 msec ON + 97 msec OFF). After electroporation, the zygotes were transferred into pseudopregnant female ICR mice and 67 mice were then delivered from a total of 230 zygotes. The two founder mice were finally obtained and confirmed by PCR, before sequencing the genomic DNA to identify the 5' loxP and 3' loxP sites. Subsequently, the founder mice were then mated with C57BL/6N mice to obtain Bob1$^{fl/+}$ mice (line #5), which exhibited the loxP insertion in cis orientation as assessed by genotyping. Next, Bob1$^{fl/+}$ mice were

crossed with $CD4^{Cre/+}$ mice to establish $CD4^{Cre/+}Bob1^{fl/fl}$ mice. To generate $FoxP3^{GFP/DTR}CD4^{Cre/+}Bob1^{fl/fl}$ mice, $CD4^{Cre/+}Bob1^{fl/fl}$ mice were mated with $FoxP3^{GFP/DTR}$ mice, which are $Foxp3^{DTR}$ knock-in mice containing an internal ribosome entry site, a human diphtheria toxin receptor (DTR), and an enhanced green fluorescent protein in the 3' UTR of the *Foxp3* locus.

(b) Representative results of PCR-based genotyping for conditional Bob1-deficient mice (Supplementary Fig. 12). The following primer pairs were used: forward primer (5'-GCTGGTTAGTGAGGCTTG-3') and reverse primer (5'-ATGAGGTCAAGGATCAGC-3') for the 5' loxP site (expected length of 223 bp and 263 bp in wild-type and mutant alleles, respectively), and forward primer (5'-TCACGTCTTGGCTGCAGG-3') and reverse primer (5'-CTGACAGCTTCACCAAGG-3') for the 3' loxP site (expected length of 413 bp and 453 bp in wild-type and mutant alleles, respectively).

(c) Immunoblotting analysis of the expression of Bob1 in B cells ($B220^{+}$) and in vitro activated $CD4^{+}$ T cells derived from $CD4^{+/+}Bob1^{fl/fl}$ and $CD4^{Cre/+}Bob1^{fl/fl}$ mice. Immunoblot analysis was independently performed to detect signals for Bob1 and β-actin due to their similar molecular weights. Merged band images from these separate immunoblots are displayed (Supplementary Fig. 12).

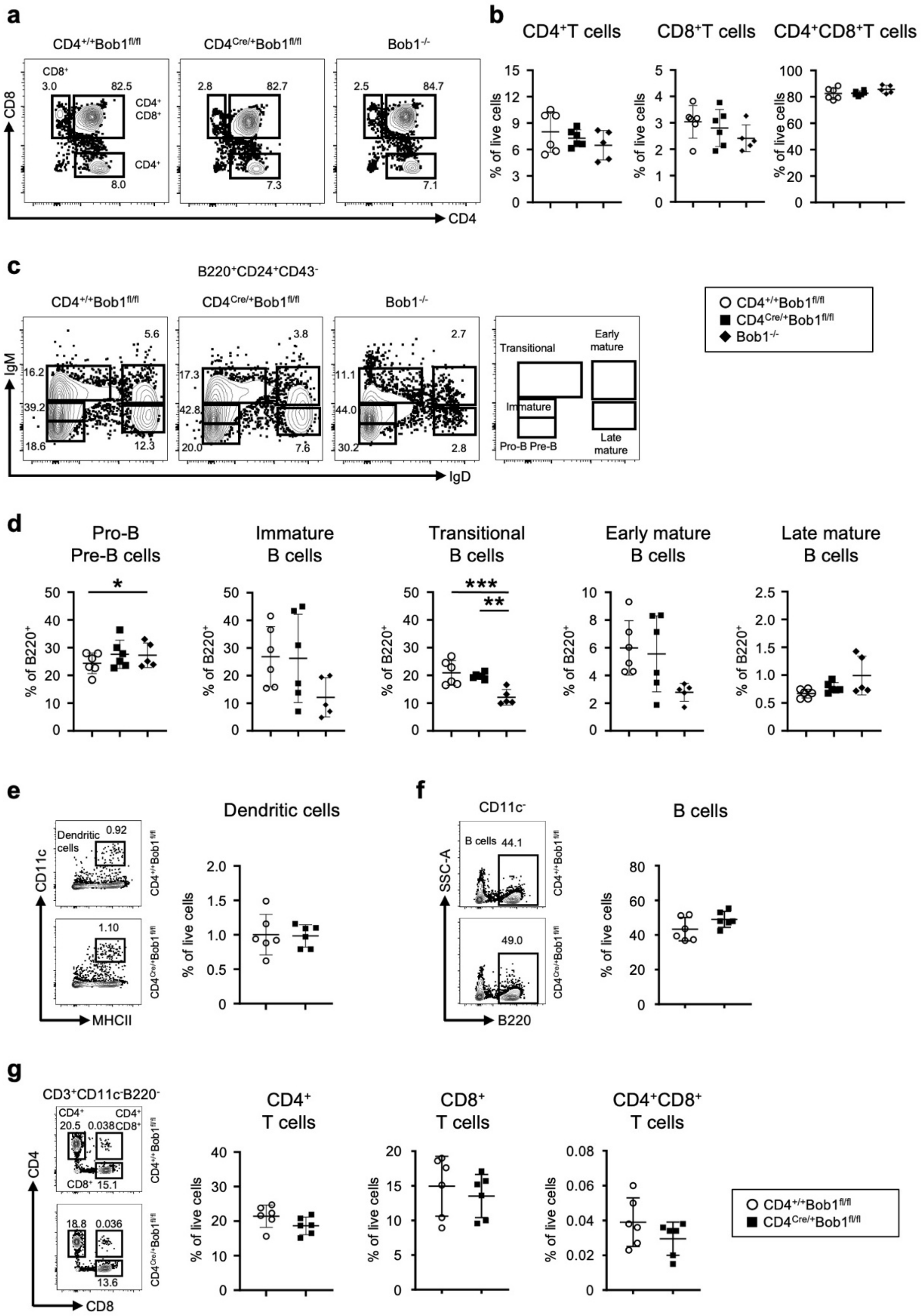

Supplementary Fig. 2. Cell populations in central and peripheral immune tissues from preimmunized CD4$^{+/+}$Bob1$^{fl/fl}$ and CD4$^{Cre/+}$Bob1$^{fl/fl}$ mice compared to those from Bob1$^{-/-}$ mice

(a, b) Flow cytometric analysis of thymocytes and quantification of T cell populations.

(a) Representative profiles of thymocytes. The ratios (%) of $CD4^+$ T cells ($CD4^+CD8^-$), $CD8^+$ T cells ($CD4^-CD8^+$), and $CD4^+CD8^+$ T cells in the total live cells are shown for each mouse strain.
(b) Graphs of thymocyte populations in each mouse group (n = 5–6).
(c, d) Flow cytometric analysis of developing B cells ($B220^+CD24^+CD43^-$) in the bone marrow and quantification of B cell populations.
(c) Representative profiles of developing B cells. The ratios (%) of IgD^-IgM^- (pro-pre) cells, IgD^-IgM^{lo} (immature) cells, IgD^-IgM^+ (transitional) cells, IgD^+IgM^+ (early mature) cells, and $IgD^+IgM^{lo/-}$ (late mature) cells in $B220^+$ B cells are shown for each mouse strain.
(d) Graphs of developing B cell populations in each mouse group (n = 5–6). Pro-B Pre-B cells, $^*P = 0.0453$, one-way ANOVA (Tukey’s multiple comparisons test). Transitional B cells, $^{***}P = 0.0010$, $^{**}P = 0.0031$, one-way ANOVA (Tukey’s multiple comparisons test).
(e-g) Flow cytometric analysis of spleen cells and quantification of immune cell populations.
(e) Representative profiles of dendritic cells (left panel). The ratios (%) of $CD11c^+$MHC Class II $(MHC\text{-}II)^+$ cells among the total live cells are shown for each mouse strain. Graphs of dendritic cells in each mouse group (n = 6) (right panel).
(f) Representative profiles of peripheral B cells (left panel). The ratios (%) of $B220^+$ B cells among the total live cells are shown for each mouse strain. Graphs of B cells in each mouse group (n = 6) (right panel).
(g) Representative profiles of peripheral T cells (left panel). The ratios (%) of $CD4^+$ T cells ($CD4^+CD8^-$), $CD8^+$ T cells ($CD4^-CD8^+$), and $CD4^+CD8^+$ T cells among the total live cells are shown for each mouse strain. Graphs of peripheral T cells in each mouse group (n = 6) (right panel).
Data are shown as the mean ± SD of each group of mice. Similar results were obtained across two or three independent experiments.

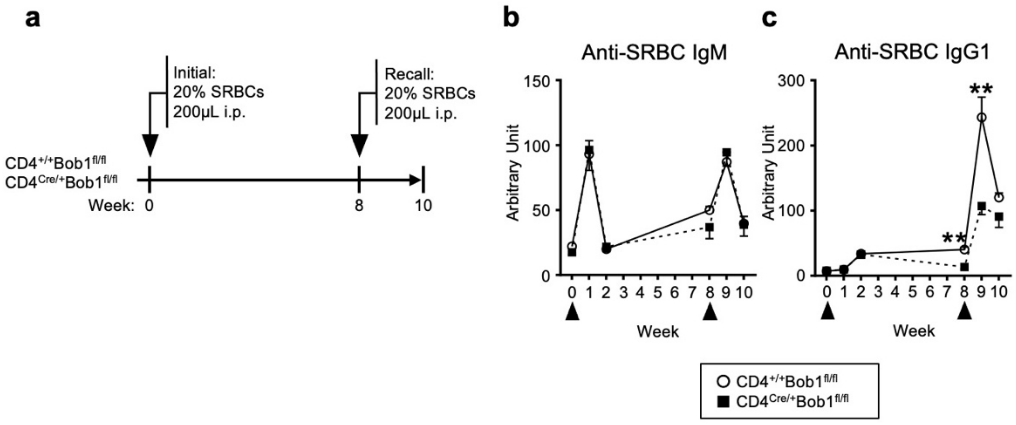

Supplementary Fig. 3. Humoral responses against SRBCs in CD4$^{+/+}$Bob1$^{fl/fl}$ and CD4$^{Cre/+}$Bob1$^{fl/fl}$ mice

(a) Experimental protocol for immunization using SRBCs. SRBCs were intraperitoneally injected at day 0 and day 57. Sera were collected from the tail vein every week until day 70.

(b, c) Time courses of the amounts of anti-SRBC-specific IgM and anti-SRBC-specific IgG1 (arbitrary units) are shown in **(b)** and **(c)**, respectively. ***P* = 0.0057 (8 week), ***P* = 0.0027 (9 week), Mann-Whitney *U*-test.

Data in (b, c) are presented as the mean ± SEM of each group of mice (n = 6–8); CD4$^{+/+}$Bob1$^{fl/fl}$ mice (open circle) and CD4$^{Cre/+}$Bob1$^{fl/fl}$ mice (closed square). Similar results were obtained across two or three independent experiments.

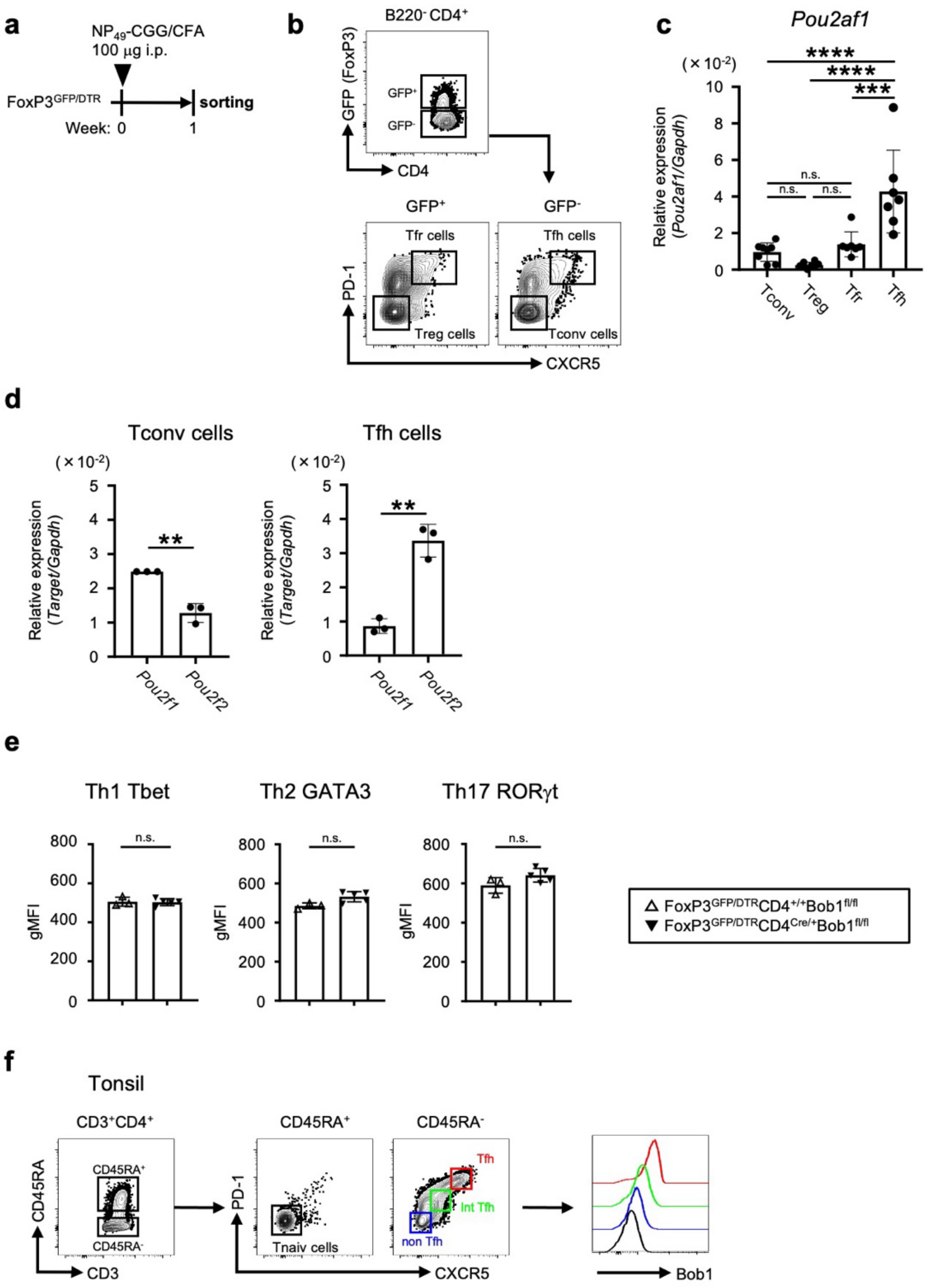

Supplementary Fig. 4. Bob1 expression in Tfh cells and $CD4^+$ T cell subsets

(a-c) Expression of Bob1 in $CD4^+$ T cell subsets of mouse splenocytes.

(a) Experimental protocol for the immunization of $FoxP3^{GFP/DTR}$ mice by NP_{49}-CGG with CFA.

(b) Sorting strategy of Tfh cells ($B220^-GFP^-CD3^+CD4^+PD\text{-}1^+CXCR5^+$), Tfr cells ($B220^-GFP^+CD3^+CD4^+CXCR5^+PD\text{-}1^+$), regulatory T (Treg cells; $B220^-GFP^+CD3^+CD4^+CXCR5^-PD\text{-}1^-$), and conventional T cells (T conv cells, $B220^-GFP^-CD3^+CD4^+CXCR5^-PD\text{-}1^-$) from the splenocytes of $FoxP3^{GFP/DTR}$ mice 1 week after the immunization shown in (a).

(c) Expression of *Pou2af1* (Bob1) in $CD4^+$ T cell subsets assessed by RT-qPCR. A dot

represents the average value of a group of mice (n = 5). ****$P < 0.0001$ (Tconv cells vs. Tfh cells), ****$P < 0.0001$ (Treg cells vs. Tfh cells), ***$P = 0.0005$ (Tfr cells vs. Tfh cells), one-way ANOVA (Tukey's multiple comparisons test). Similar results were obtained across two independent experiments.

(d) Expressions of *Pou2f1* (Oct-1) and *Pou2f2* (Oct-2) in Tconv and Tfh cells (left and right panels, respectively), as assessed by RT-qPCR. A dot represents the average value of a group of mice (n = 5). **$P = 0.0016$ (Tconv cells; *Pou2f1* vs. *Pou2f2)*, **$P = 0.0012$ (Tfh cells; *Pou2f1* vs. *Pou2f2*); unpaired *t*-test. Similar results were obtained across three independent experiments.

(e) *In vitro* differentiation experiments using naïve $CD4^+$ T cells ($B220^-GFP^-CD4^+CD44^-CD62L^+$) purified from the splenocytes of preimmune $FoxP3^{GFP/DTR}CD4^{+/+}Bob1^{fl/fl}$ and $FoxP3^{GFP/DTR}CD4^{Cre/+}Bob1^{fl/fl}$ mice. Graphs show the expression levels of Tbet, GATA3, and RORγt in cells under specific conditions for Th1 cells, Th2 cells, and Th17 cells, respectively, assessed by intracellular flow cytometry with the geometric mean fluorescence intensity index (gMFI). Individual data in graphs were obtained from triplicates in a single experiment. Similar results were obtained across three independent experiments.

(f) Expression of Bob1 in Tfh cells from human tonsils assessed by intracellular flow cytometry. Tfh cells ($CD3^+CD4^+CD45RA^-CXCR5^+PD\text{-}1^+$, red) had a high expression of Bob1, and int-Tfh cells ($CD3^+CD4^+CD45RA^-CXCR5^{lo}PD\text{-}1^{lo}$, green) expressed Bob1 to a lesser extent compared to non-Tfh cells ($CD3^+CD4^+CD45RA^-CXCR5^-PD\text{-}1^-$, blue) and naïve $CD4^+$ T cells ($CD3^+CD4^+CD45RA^+CXCR5^-PD\text{-}1^-$, black). Similar results were obtained across three independent experiments.

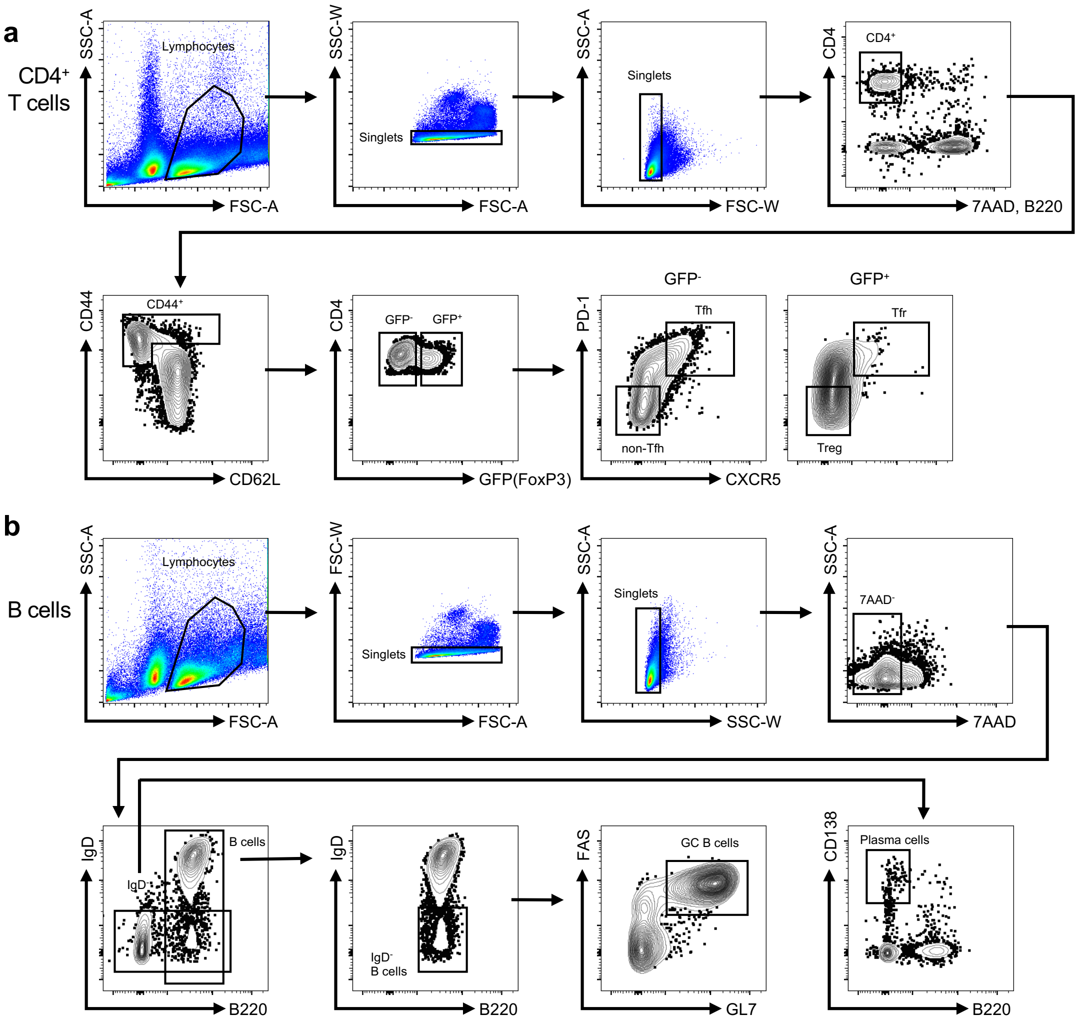

Supplementary Fig. 5. Gating strategy and flow cytometric analysis of tissue-resident lymphocytes.

Gating strategy of lymphocytes in FoxP3$^{GFP/DTR}$CD4$^{+/+}$Bob1$^{fl/fl}$ and FoxP3$^{GFP/DTR}$CD4$^{Cre/+}$Bob1$^{fl/fl}$ mice.

(a) CD4^{+} T cell subsets.

(b) B cell subsets.

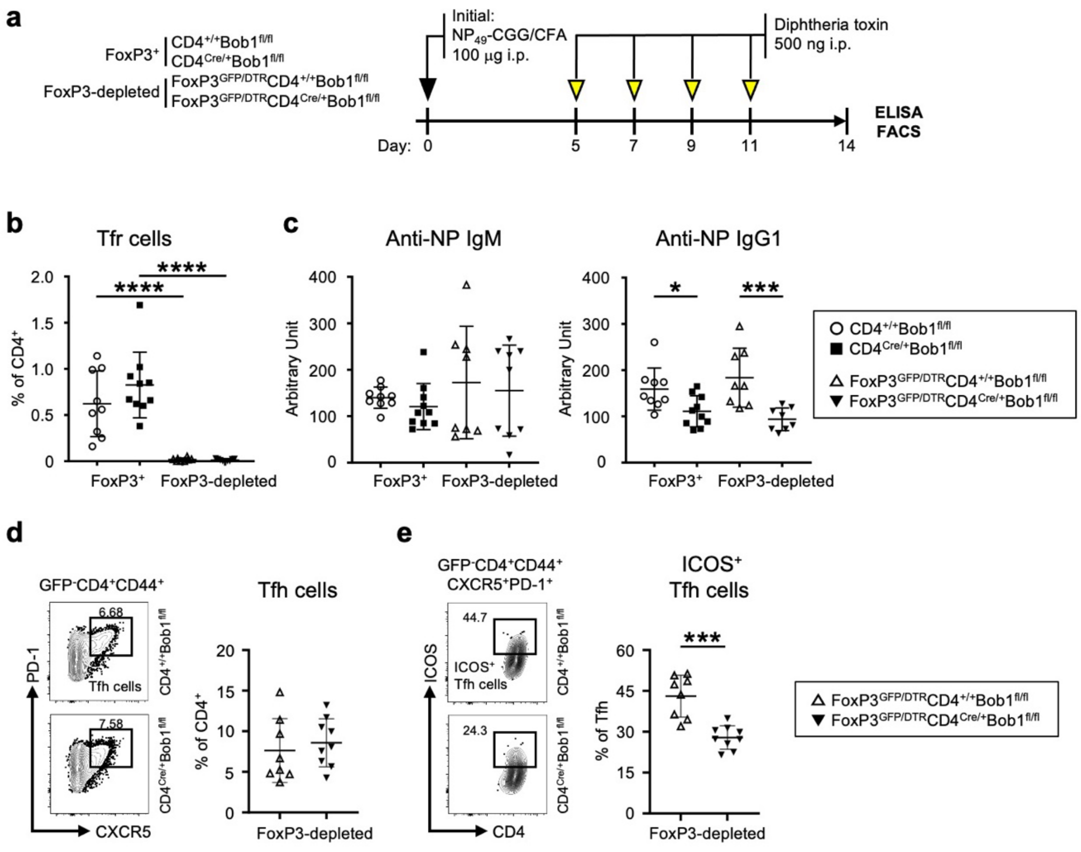

Supplementary Fig. 6. Bob1 regulates Tfh cells regardless of Tfr cells

(a) Experimental protocol for immunization and subsequent *in vivo* depletion of Tfr cells. NP_{49}-CGG with CFA was administered intraperitoneally on day 0. Then, diphtheria toxin was administered intraperitoneally on days 5, 7, 9, and 11, and the sera and spleen cells were analyzed on day 14. Following this protocol, $FoxP3^{GFP/DTR}CD4^{Cre/+}Bob1^{fl/fl}$ and $FoxP3^{GFP/DTR}CD4^{+/+}Bob1^{fl/fl}$, and $CD4^{Cre/+}Bob1^{fl/fl}$ and $CD4^{+/+}Bob1^{fl/fl}$ mice were analyzed.

(b) The depletion of Tfr cells in $FoxP3^{GFP/DTR}CD4^{Cre/+}Bob1^{fl/fl}$ and $FoxP3^{GFP/DTR}CD4^{+/+}Bob1^{fl/fl}$ mice was induced by treatment with diphtheria toxin. ****$P < 0.0001$.

(c) Serum levels of NP-specific IgM and IgG1 as assessed by ELISA using NP_{23}-BSA. NP-specific IgG1, *$P = 0.0172$, ***$P = 0.0011$.

(d, e) Representative flow cytometric profiles and graphs of Tfh cells in spleens from $FoxP3^{GFP/DTR}CD4^{Cre/+}Bob1^{fl/fl}$ and $FoxP3^{GFP/DTR}CD4^{+/+}Bob1^{fl/fl}$ mice.

(d) Profiles of Tfh cells ($GFP^-CD4^+CD44^+CXCR5^+PD\text{-}1^+$) and graphs showing the % of Tfh cells among the total $CD4^+$ T cells.

(e) Profiles of $ICOS^+$ Tfh cells and graphs showing the % of $ICOS^+$ Tfh cells among the total Tfh cells. ***$P = 0.0003$.

Data represent the mean ± SD of 8–10 mice per group. Statistical significance was analyzed by the Mann-Whitney *U*-test; *$P < 0.05$, ***$P < 0.001$, ****$P < 0.0001$. Data shown in (b, c) are from $CD4^{+/+}Bob1^{fl/fl}$ mice (open circle) and $CD4^{Cre/+}Bob1^{fl/fl}$ mice (closed square), and data shown in (b-e) are from $FoxP3^{GFP/DTR}CD4^{+/+}Bob1^{fl/fl}$ mice

(open triangle) and FoxP3$^{GFP/DTR}$CD4$^{Cre/+}$Bob1$^{fl/fl}$ mice (closed triangle). Similar results were obtained across three independent experiments.

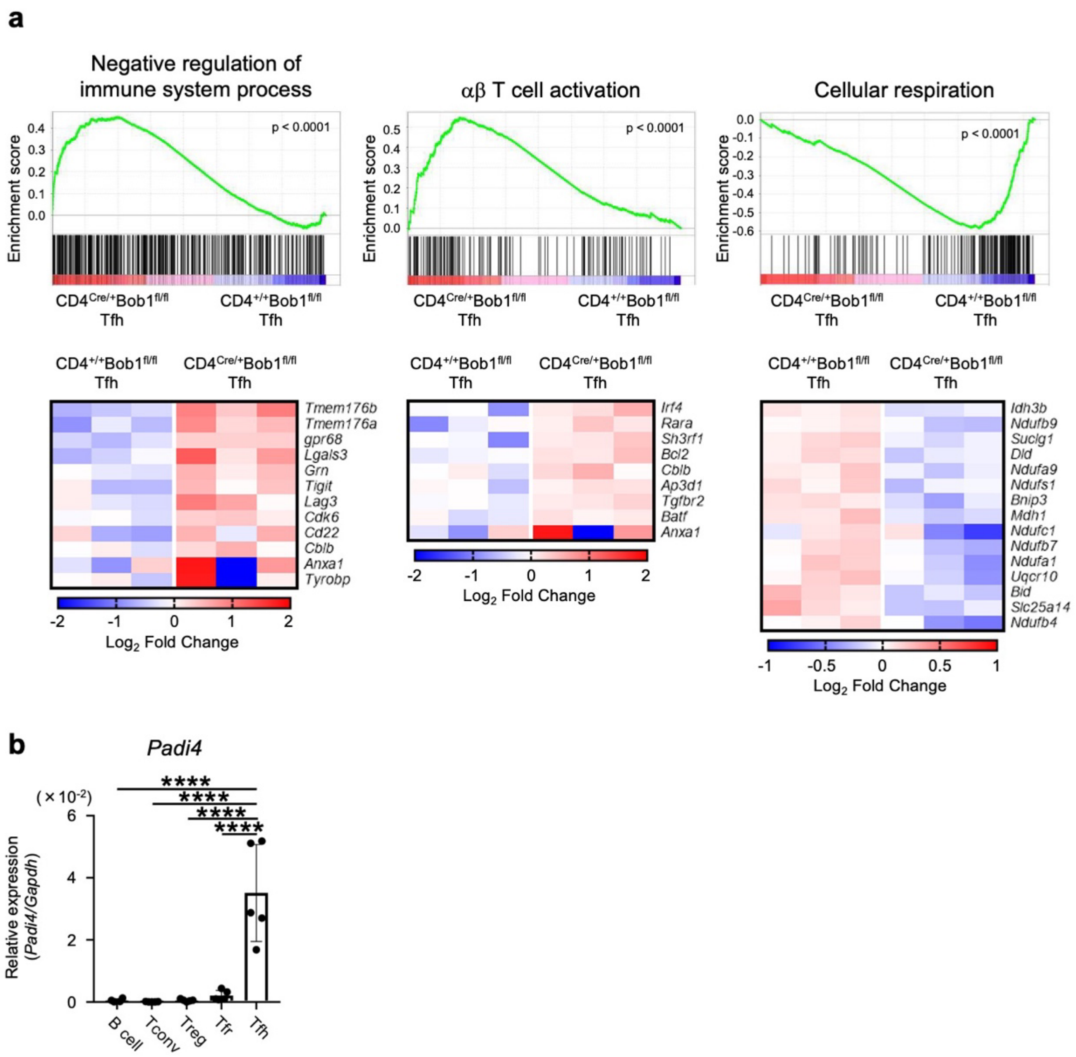

Supplementary Fig. 7. GSEA and a corresponding heat map of the transcriptomes of $CD4^{+/+}Bob1^{fl/fl}$ and $CD4^{Cre/+}Bob1^{fl/fl}$ Tfh cells

(a) Graphical representations of GSEA and heat maps of the corresponding rank-ordered genes are shown in the upper and lower panels, respectively, based on the RNAseq analysis of Tfh cells from $FoxP3^{GFP/DTR}CD4^{+/+}Bob1^{fl/fl}$ and $FoxP3^{GFP/DTR}CD4^{Cre/+}Bob1^{fl/fl}$ mice (Fig. 3). GSEA showing that downregulated genes in $CD4^{Cre/+}Bob1^{fl/fl}$ Tfh cells are related to cellular respiration (GO: 0045333) as shown in heat maps. GSEA analyses show genes related to negative regulators of the immune system process (GO: 0002683) and αβ-T cell activation (GO: 0046631) are upregulated in $CD4^{Cre/+}Bob1^{fl/fl}$ Tfh cells as shown in heat maps. Relative values of gene expression are indicated by color in each panel. In all GSEA, nominalized *P*-values are < 0.0001.

(b) Expression of *Padi4* in $CD4^+$ T cell subsets and B cells of $FoxP3^{GFP/DTR}$ mouse splenocytes obtained after immunization. *****P* < 0.0001, one-way ANOVA (Tukey's multiple comparisons test).

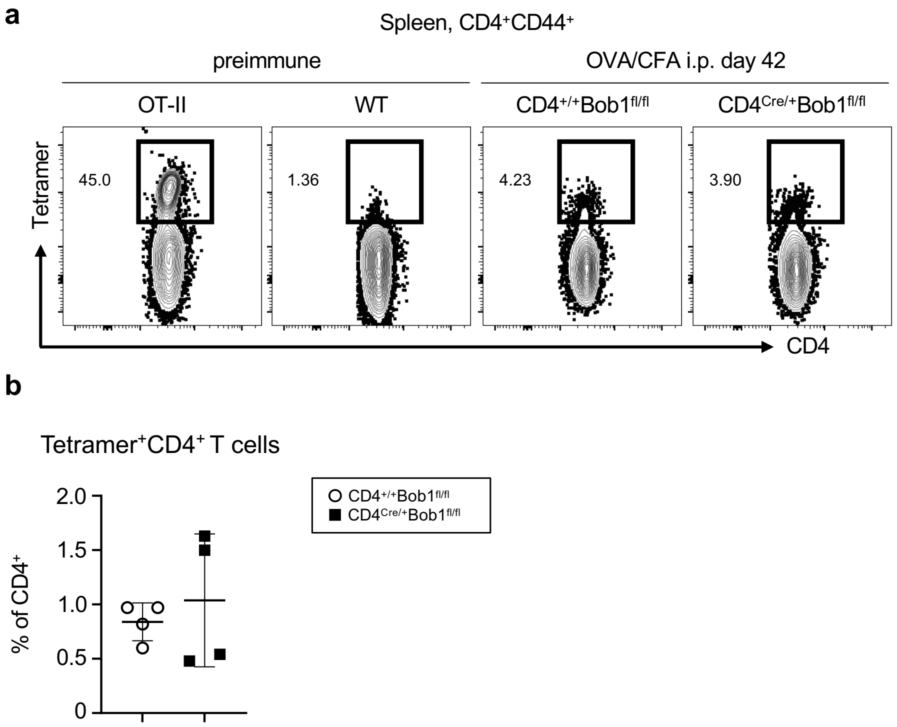

Supplementary Fig. 8. Analysis of OVA-specific CD4^{+} T cells (corresponding to Fig. 4)

(a) Representative flow cytometric profiles of I-A^{b} OVA$_{323\text{-}339}$ tetramer-positive CD4^{+} T cells.

(b) Graphs showing Tetramer^{+}CD4^{+} T cells as a percentage of the total CD4^{+} T cells. Data represent the mean ± SD of 4 mice per group. Statistical significance was determined using the Mann-Whitney *U*-test. Similar results were obtained in three independent experiments.

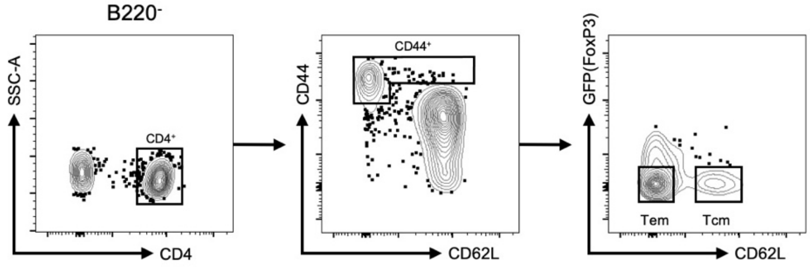

Supplementary Fig. 9. Gating strategy of Tcm and Tem cells in adoptive cell transfer analysis

Gating strategy for the sorting of splenic Tcm and Tem cells from FoxP3$^{GFP/DTR}$CD4$^{+/+}$Bob1$^{fl/fl}$ and FoxP3$^{GFP/DTR}$CD4$^{Cre/+}$Bob1$^{fl/fl}$ mice.

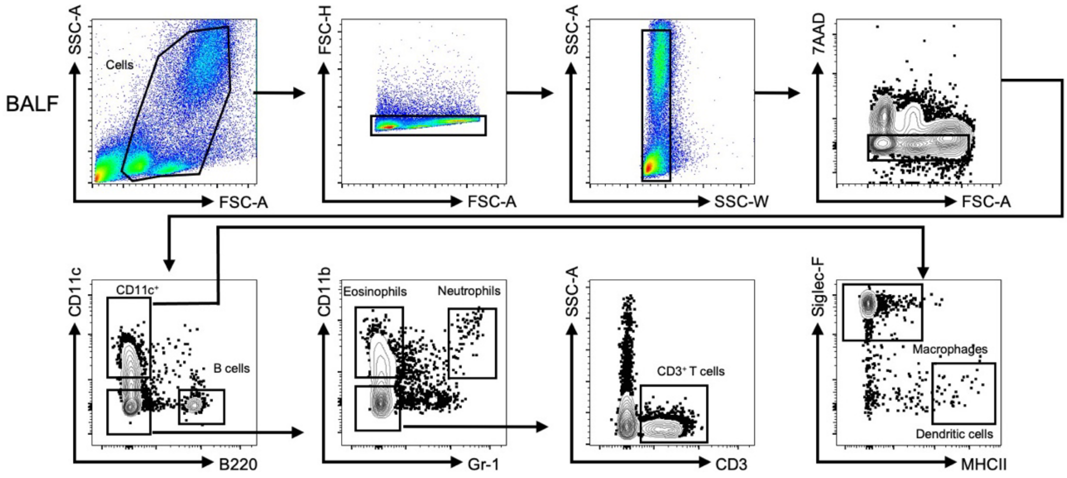

Supplementary Fig. 10. Gating strategy and flow cytometric analysis of HDM-induced allergic asthma models

Gating strategy of inflammatory and immune cells in BALF of $FoxP3^{GFP/DTR}CD4^{+/+}Bob1^{fl/fl}$ and $FoxP3^{GFP/DTR}CD4^{Cre/+}Bob1^{fl/fl}$ mice.

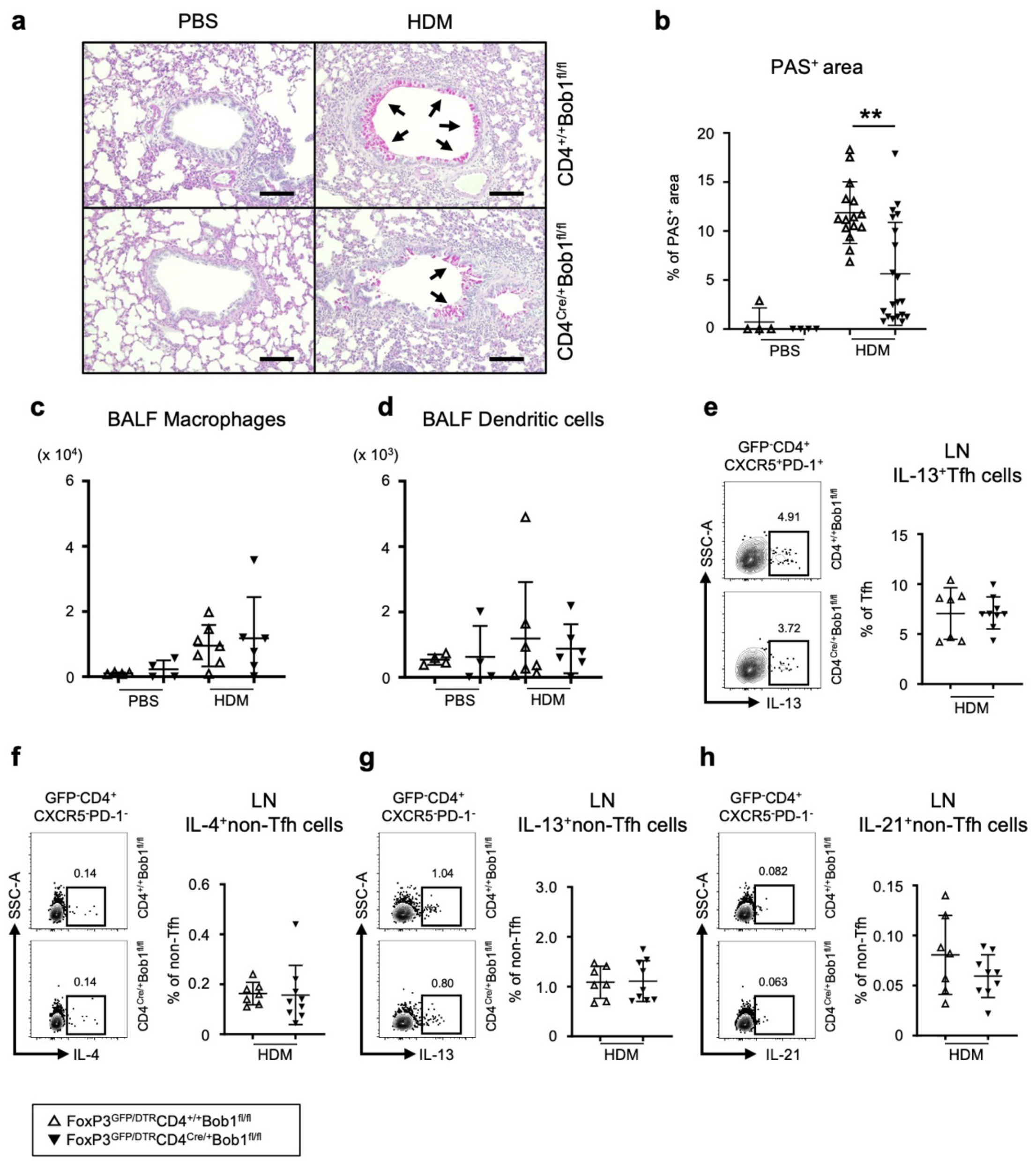

Supplementary Fig. 11. Analysis of HDM-induced allergic asthma models (corresponding to Fig. 6)

(a) Representative microscopy images of formalin-fixed paraffin-embedded tissue sections of inflamed lung lesions on day 37 stained with PAS. Tissue sections are corresponding to those in Fig. 6b. Arrows indicate mucous production by airway goblet cells. Scale bar: 100 μm.

(b) Quantification of PAS-positive mucous cells in airways. Graphs depict the ratio of PAS$^+$ areas within the airway area for each group. ***P* = 0.0012.

(c) Number of macrophages (CD11c$^+$Siglec-F$^+$MHCII^{lo}) in the BALF.

(d) Number of dendritic cells (CD11c$^+$Siglec-F$^-$MHCII^{hi}) in the BALF.

(e) Representative flow cytometric profiles for the expression of IL-13 in Tfh cells (GFP$^-$B220$^-$CD4$^+$CXCR5$^+$PD-1$^+$) residing in the mediastinal lymph nodes. Graphs display IL-13$^+$ Tfh cells (Tfh13 cells).

(f-h) Representative flow cytometric profiles of the expressions of cytokines in non-Tfh cells ($GFP^-B220^-CD4^+CXCR5^-PD\text{-}1^-$) residing in the mediastinal lymph nodes. Graphs show cytokine-expressing non-Tfh cells: **(f)** IL-4, **(g)** IL-13, and **(h)** IL-21.
Data shown in (b-h) represent the mean ± SD of each group of mice (n = 4–9). Data from $FoxP3^{GFP/DTR}CD4^{+/+}Bob1^{fl/fl}$ and $FoxP3^{GFP/DTR}CD4^{Cre/+}Bob1^{fl/fl}$ mice are shown as mice open and closed triangles, respectively. Statistical significance was analyzed by the Mann-Whitney *U*-test; $^{**}P < 0.01$. Similar results were obtained across two to three independent experiments.

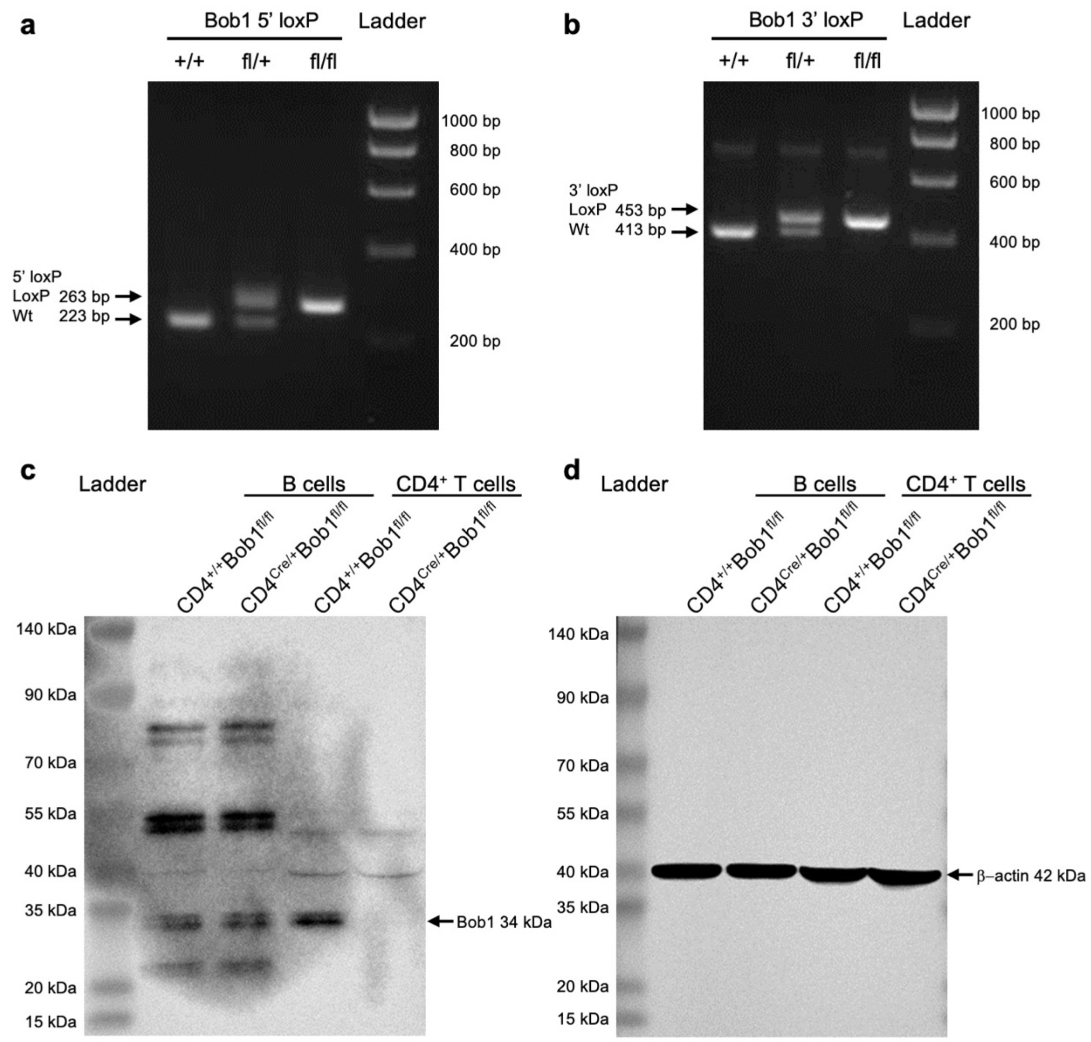

Supplementary Fig. 12. Original images of gel electrophoresis for PCR products and immunoblot analysis.

(a-b) Original images displaying gel electrophoresis results of PCR products as depicted in Supplementary Fig. 1b.

(a) 5' loxP site with expected lengths of 223 bp and 263 bp for wild-type and mutant alleles, respectively.

(b) 3' loxP site with expected lengths of 413 bp and 453 bp for wild-type and mutant alleles, respectively.

(c-d) Original images of immunoblot analysis as presented in Supplementary Fig. 1c.

(c) Bob1 with expected molecular weight of 34 kDa.

(d) β-actin with expected molecular weight of 42 kDa.
