# Supplementary material for: Bob1 maintains T follicular helper cells for long-term humoral immunity
Source: Commun Biol. 2024 Feb 15;7:185. doi: 10.1038/s42003-024-05827-0 (PMC10869348; doi:10.1038/s42003-024-05827-0)
Supplement: Supplementary file 2 — Description of Additional Supplementary Files [file 42003_2024_5827_MOESM2_ESM.pdf]

Description of Additional Supplementary Files

File name: Supplementary Data
Description: Numerical source data for the graphs in the main and supplementary figures.
