# Supplementary material for: Bob1 maintains T follicular helper cells for long-term humoral immunity
Source: Commun Biol. 2024 Feb 15;7:185. doi: 10.1038/s42003-024-05827-0 (PMC10869348; doi:10.1038/s42003-024-05827-0)
Supplement: Supplementary file 4 — Reporting Summary [file 42003_2024_5827_MOESM4_ESM.pdf]

nature portfolio

Corresponding author(s): Shingo Ichimiya

Last updated by author(s): Oct 30, 2023

Reporting Summary

Nature Portfolio wishes to improve the reproducibility of the work that we publish. This form provides structure for consistency and transparency in reporting. For further information on Nature Portfolio policies, see our Editorial Policies and the Editorial Policy Checklist.

Statistics

For all statistical analyses, confirm that the following items are present in the figure legend, table legend, main text, or Methods section.

n/a	Confirmed	
☐	☒	The exact sample size (*n*) for each experimental group/condition, given as a discrete number and unit of measurement
☐	☒	A statement on whether measurements were taken from distinct samples or whether the same sample was measured repeatedly
☐	☒	The statistical test(s) used AND whether they are one- or two-sided<br>*Only common tests should be described solely by name; describe more complex techniques in the Methods section.*
☒	☐	A description of all covariates tested
☒	☐	A description of any assumptions or corrections, such as tests of normality and adjustment for multiple comparisons
☐	☒	A full description of the statistical parameters including central tendency (e.g. means) or other basic estimates (e.g. regression coefficient) AND variation (e.g. standard deviation) or associated estimates of uncertainty (e.g. confidence intervals)
☐	☒	For null hypothesis testing, the test statistic (e.g. *F*, *t*, *r*) with confidence intervals, effect sizes, degrees of freedom and *P* value noted<br>*Give P values as exact values whenever suitable.*
☒	☐	For Bayesian analysis, information on the choice of priors and Markov chain Monte Carlo settings
☒	☐	For hierarchical and complex designs, identification of the appropriate level for tests and full reporting of outcomes
☒	☐	Estimates of effect sizes (e.g. Cohen's *d*, Pearson's *r*), indicating how they were calculated

Our web collection on statistics for biologists contains articles on many of the points above.

Software and code

Policy information about availability of computer code

Data collection	Flow cytometry: BD FACS Diva Software (v8.0.1)<br>RNA sequencing: NovaSeq Control Software (v1.7)<br>RT-qPCR: Lightcyler96 (v1.01.01.0050)<br>Laser confocal microscopy: ZEN black edition (v2.3.SP1.FP3)<br>Western blotting: ImageSaver6 (v2.7.1)<br>Extracellular flux assay: Wave (v2.6.3.5)
Data analysis	Flow cytometry: FlowJo (v10.8.1)<br>RNA sequence data analysis: Cutadapt (v1.2.1), PRINSEQ (v0.19.2), TopHat (v2.0.13), Cufflinks (v2.2.1), ggVolcanoR (v1.0), GSEA (v4.1.0)<br>Laser confocal microscopy images: ZEN blue edition (v3.4)<br>Extracellular flux assay: Wave (v2.6.3.5)<br>Statistical analysis: GraphPad Prism (v7.0)

For manuscripts utilizing custom algorithms or software that are central to the research but not yet described in published literature, software must be made available to editors and reviewers. We strongly encourage code deposition in a community repository (e.g. GitHub). See the Nature Portfolio guidelines for submitting code & software for further information.

Data

Policy information about availability of data

All manuscripts must include a data availability statement. This statement should provide the following information, where applicable:

- Accession codes, unique identifiers, or web links for publicly available datasets
- A description of any restrictions on data availability
- For clinical datasets or third party data, please ensure that the statement adheres to our policy

The authors declare that the main data supporting the findings of this study are available within the article and its Supplementary Information. RNAseq data have been deposited in the Gene Expression Omnibus (accession number GSE223725).

Human research participants

Policy information about studies involving human research participants and Sex and Gender in Research.

Reporting on sex and gender	This study did not contain sex and gender specific analysis.
Population characteristics	Tonsil samples for the analysis of lymphocytes were acquired from tonsillectomy cases with tonsillar hypertrophy or recurrent tonsillitis .
Recruitment	The criteria of the subjects in this study was based on clinical diagnosis of tonsillar hypertrophy or recurrent tonsillitis.
Ethics oversight	All experiments using human samples were performed in accordance with the study protocols approved by the Institutional Review Board of Sapporo Medical University.

Note that full information on the approval of the study protocol must also be provided in the manuscript.

Field-specific reporting

Please select the one below that is the best fit for your research. If you are not sure, read the appropriate sections before making your selection.

☒ Life sciences ☐ Behavioural & social sciences ☐ Ecological, evolutionary & environmental sciences

For a reference copy of the document with all sections, see nature.com/documents/nr-reporting-summary-flat.pdf

Life sciences study design

All studies must disclose on these points even when the disclosure is negative.

Sample size	Sample size was chosen in each case according to experimental design. Based on empirical data, previous publications and availability of mice, sample sizes were selected so as to power statistical analyses.
Data exclusions	No data were excluded from the analysis.
Replication	All experiments were performed multiple independent times per condition. All experiments were shown to be reproducible.
Randomization	Allocation into experimental groups was randomized.
Blinding	Blinding was not relevant to our study design. The samples were prepared, treated, and analyzed by the same standard procedure in each experiment.

Reporting for specific materials, systems and methods

We require information from authors about some types of materials, experimental systems and methods used in many studies. Here, indicate whether each material, system or method listed is relevant to your study. If you are not sure if a list item applies to your research, read the appropriate section before selecting a response.

Materials & experimental systems

n/a	Involved in the study
☐	☒ Antibodies
☒	☐ Eukaryotic cell lines
☒	☐ Palaeontology and archaeology
☐	☒ Animals and other organisms
☒	☐ Clinical data
☒	☐ Dual use research of concern

Methods

n/a	Involved in the study
☒	☐ ChIP-seq
☐	☒ Flow cytometry
☒	☐ MRI-based neuroimaging

Antibodies

Antibodies used

Antibodies for analyzing human samples;
CD3e FITC (SK7 #349201 BD Biosciences dilution 1/100)
CD4 APC-Cy7 (RPA-T4 #557871 BD Biosciences dilution 1/100)
CD45RA BV510 (HI100 #304142 BioLegend dilution 1/100)
CD185 / CXCR5 PerCP-Cy5.5 (RF8B2 #562781 BD Biosciences dilution 1/100)
CD279 / PD-1 PE (EH12.1 #560795 BD Biosciences dilution 1/100)
Bob1 (C20 #sc-23932 SantaCruz dilution 1/100)
Donkey Anti-Rabbit IgG BV421 (Poly4064 #406410 BioLegend dilution 1/100)

Antibodies for analyzing mouse samples;
CD3e Purified (145-2C11 #100302 BioLegend Dilution 1/100)
CD3e APC-Cy7 (145-2C11 Cat#100330 BioLegend Dilution 1/200)
CD3e PE-Cy7 (145-2C11 Cat#552774 BD Biosciences Dilution 1/200)
CD4 APC-Cy7 (GK1.5 Cat#100414 BioLegend Dilution 1/200)
CD4 BV510 (RM4-5 Cat#563106 BD Biosciences Dilution 1/200)
CD8a PE (53-6.7 Cat#553032 BD Biosciences Dilution 1/200)
CD8a BV510 (53-6.7 Cat#563068 BD Biosciences Dilution 1/200)
CD11c PE (N418 Cat#565592 BD Biosciences Dilution 1/200)
CD11c PerCP-Cy5.5 (N418 Cat#117328 BioLegend Dilution 1/200)
CD11b BB515 (M1/70 Cat#564455 BD Biosciences Dilution 1/200)
CD16/32 Purified (2.4G2 Cat#553142 BD Biosciences Dilution 1/200)
CD24 Biotin (M1/69 Cat#553260 BD Biosciences Dilution 1/200)
CD28 Purified (37.51 Cat#102102 BioLegend Dilution 1/330)
CD38 APC (90 Cat#102712 BioLegend Dilution 1/200)
CD43 PE (S7 Cat#553271 BD Biosciences Dilution 1/200)
CD44 PE-Cy7 (IM7 Cat#103030 BioLegend Dilution 1/200)
CD45.1 AF488 (A20 Cat#110717 BioLegend Dilution 1/200)
CD45.1 APC-Cy7 (A20 Cat#110715 BioLegend Dilution 1/200)
CD45.2 PE-Cy7 (104 Cat#560696 BD Biosciences Dilution 1/200)
CD45R / B220 PerCP-Cy5.5 (RA3-6B2 Cat#552771 BD Biosciences Dilution 1/200)
CD45R / B220 APC (RA3-6B2 Cat#553092 BD Biosciences Dilution 1/200)
CD45R / B220 BV510 (RA3-6B2 Cat#103248 BioLegend Dilution 1/200)
CD62L APC (MEL-14 Cat#104412 BioLegend Dilution 1/200)
CD62L APC-Cy7 (MEL-14 Cat#560514 BD Biosciences Dilution 1/200)
CD95 / Fas PE-Cy7 (Jo2 Cat#557653 BD Biosciences Dilution 1/200)
CD138 / Syndecan-1 APC-Cy7 (281-2 Cat#142529 BioLegend Dilution 1/200)
CD170 / Siglec-F BV421 (E50-2440 Cat#565934 BD Biosciences Dilution 1/200)
CD185 / CXCR5 PE (L138D7 Cat#145504 BioLegend Dilution 1/100)
CD185 / CXCR5 APC-Cy7 (L138D7 Cat#145526 BioLegend Dilution 1/100)
CD185 / CXCR5 BV421 (L138D7 Cat#145512 BioLegend Dilution 1/100)
CD223 / LAG3 APC (C9B7W Cat#125209 BioLegend Dilution 1/100)
CD278 / ICOS PE (7E.17G9 Cat#117405 BioLegend Dilution 1/100)
CD278 / ICOS BV421 (7E.17G9 Cat#564070 BD Biosciences Dilution 1/100)
CD279 / PD-1 APC (29F.1A12 Cat#135210 BioLegend Dilution 1/200)
CD279 / PD-1 PE-Cy7 (9F.1A12 Cat#135215 BioLegend Dilution 1/200)
FR4 APC-Fire750 (12A5 Cat#125013 BioLegend Dilution 1/200)
TIGIT AF647 (GIGD7 Cat#51-9501-82 eBioscience Dilution 1/100)
Gr-1 APC (RB6-8C5 Cat#561083 BD Biosciences Dilution 1/200)
GL7 Pacific Blue (GL7 Cat#144614 BioLegend Dilution 1/200)
I-A/I-E PE-Cy7 (M5/114.15.2 Cat#107629 BioLegend Dilution 1/200)
IgM PE-Cy7 (R6-60.2 Cat#552867 BD Biosciences Dilution 1/200)
IgD FITC (11-26c.2a Cat#553439 BD Biosciences Dilution 1/200)
IgG1 FITC (A85-1 Cat#553443 BD Biosciences Dilution 1/200)
IFNg Purified (R4-6A2 Cat#505702 BioLegend Dilution 1/200)
IL-4 Purified (11B11 Cat#504102 BioLegend Dilution 1/100)
IL-4 BV421 (11B11 Cat#504119 BioLegend Dilution 1/100)
IL-13 PE-Cy7 (eBio13A Cat#25-7133-80 eBioscience Dilution 1/1l00)
Il-21 PE (FFA21 Cat#12-7211-80 eBioscience Dilution 1/100)
Ki-67 BV421 (SolA15 Cat#48-5698-80 eBioscience Dilution 1/100)

	FoxP3 FITC (FJK-16s Cat#11-5773-82 eBioscience Dilution 1/100)<br>T-bet APC (4B10 Cat#644813 BioLegend Dilution 1/100)<br>GATA3 PE (L50-823 Cat#560074 BD Biosciences Dilution 1/100)<br>RORγt BV421 (Q31-378 Cat#562894 BD Biosciences Dilution 1/100)<br>NP PE (Cat#N-5070-1 Biosearch Technologies Dilution 1/100)<br>I-Ab OVA323-339 Tetramer PE (Cat#TS-M710-1 MBL Dilution 1/6)<br>7AAD (#555815 BD Biosciences Dilution 1/20)<br>Streptavidin APC-Cy7 (Cat#554063 BD Biosciences Dilution 1/200)<br>PNA AF594 (Cat#L32459 invitrogen Dilution 1/100)<br>DAPI (Cat#D1306 invitrogen Dilution 1/1000)<br>BrdU APC (Cat#51-23619L BD Biosciences Dilution 1/50)
Validation	All antibodies are established, well described, commercially available, and have been validated as noted on the websites of manufacturers.

Animals and other research organisms

Policy information about studies involving animals; ARRIVE guidelines recommended for reporting animal research, and Sex and Gender in Research

Laboratory animals	Bob1fl/+ mice (Accession No. CDB0030E) on a C57BL/6 background were established in this study. CD4Cre (B6.Cg-Tg(CD4-Cre)1Cwi/BfluJ), FoxP3GFP/DTR (B6.129Cg-Foxp3tm3(DTR/GFP)Ayr/J), OT-II (B6.Cg-Tg(TcraTcrb)425Cbn/J),TCR beta chain -/- (B6.129P2-Tcrbtm1Mom/J), Bob1-/- (B6;129-Pou2af1tm1Rgr/J) were obtained from the Jackson Laboratory. Ly5.1-congenic C57BL/6 mice (CD45.1, B6.SJL-Ptprca/Rbrc) were obtained from RIKEN Bioresource Center. C57BL/6 mice were obtained from Sankyo Laboratory. All mice were maintained in specific pathogen-free conditions in the animal facility of Sapporo Medical University.
Wild animals	No wild animals were included in this study.
Reporting on sex	Experimental groups using age- (6–12 weeks) and sex-matched mice in each group were analyzed unless otherwise stated.
Field-collected samples	No field-collected samples were included in this study.
Ethics oversight	All experimental procedures performed on mice used in this study were in accordance with guidelines and policies of Sapporo Medical University and RIKEN Center for Biosystems Dynamics Research.

Note that full information on the approval of the study protocol must also be provided in the manuscript.

Flow Cytometry

Plots

Confirm that:

☒ The axis labels state the marker and fluorochrome used (e.g. CD4-FITC).

☒ The axis scales are clearly visible. Include numbers along axes only for bottom left plot of group (a 'group' is an analysis of identical markers).

☒ All plots are contour plots with outliers or pseudocolor plots.

☒ A numerical value for number of cells or percentage (with statistics) is provided.

Methodology

Sample preparation	Single-cell suspensions from tissues and blood were prepared using a 70 micron cell strainer and density-gradient centrifugation with Lympholyte. After treatment with an anti-CD16/CD32 mAb on ice 10 min, cells were stained with fluorochrome-conjugated monoclonal antibodies in PBS supplemented with 0.5% BSA and 2 mM EDTA on ice for 1 h followed by 7AAD staining.
Instrument	BD Canto, BD Aria II, BD Aria III
Software	BD FACSDiva (data collection), FlowJo (data analysis)
Cell population abundance	Post sort analysis was done by re-running collection tubes on cell sorter.
Gating strategy	All samples were pregated on FSC-A-FSC-H singlets, FSC-A-SSC-A for lymphocyte population. Gating strategy are included in Supplementary Information.

☒ Tick this box to confirm that a figure exemplifying the gating strategy is provided in the Supplementary Information.
